# Regulation of brain fluid volumes and pressures: basic principles, intracranial hypertension, ventriculomegaly and hydrocephalus

**DOI:** 10.1186/s12987-024-00532-w

**Published:** 2024-07-17

**Authors:** Stephen B. Hladky, Margery A. Barrand

**Affiliations:** Department of Pharmacology, Tennis Court Rd, Cambridge, CB2 1PD UK

**Keywords:** Cerebrospinal fluid volume, Cerebrospinal fluid flow, Idiophathic normal pressure hydrocephalus (iNPH), Interstitial fluid pressure (*ISFP*), Idiopathic intracranial hypertension (iIH), Pulsatility, Stress versus pressure, Subarachnoid spaces, Venous sinus pressure

## Abstract

The principles of cerebrospinal fluid (CSF) production, circulation and outflow and regulation of fluid volumes and pressures in the normal brain are summarised. Abnormalities in these aspects in intracranial hypertension, ventriculomegaly and hydrocephalus are discussed. The brain parenchyma has a cellular framework with interstitial fluid (ISF) in the intervening spaces. Framework stress and interstitial fluid pressure (*ISFP*) combined provide the total stress which, after allowing for gravity, normally equals intracerebral pressure (*ICP*) with gradients of total stress too small to measure. Fluid pressure may differ from *ICP* in the parenchyma and collapsed subarachnoid spaces when the parenchyma presses against the meninges. Fluid pressure gradients determine fluid movements. In adults, restricting CSF outflow from subarachnoid spaces produces intracranial hypertension which, when CSF volumes change very little, is called idiopathic intracranial hypertension (iIH). Raised *ICP* in iIH is accompanied by increased venous sinus pressure, though which is cause and which effect is unclear. In infants with growing skulls, restriction in outflow leads to increased head and CSF volumes. In adults, ventriculomegaly can arise due to cerebral atrophy or, in hydrocephalus, to obstructions to intracranial CSF flow. In non-communicating hydrocephalus, flow through or out of the ventricles is somehow obstructed, whereas in communicating hydrocephalus, the obstruction is somewhere between the cisterna magna and cranial sites of outflow. When normal outflow routes are obstructed, continued CSF production in the ventricles may be partially balanced by outflow through the parenchyma via an oedematous periventricular layer and perivascular spaces. In adults, secondary hydrocephalus with raised *ICP* results from obvious obstructions to flow. By contrast, with the more subtly obstructed flow seen in normal pressure hydrocephalus (NPH), fluid pressure must be reduced elsewhere, e.g. in some subarachnoid spaces. In idiopathic NPH, where ventriculomegaly is accompanied by gait disturbance, dementia and/or urinary incontinence, the functional deficits can sometimes be reversed by shunting or third ventriculostomy. Parenchymal shrinkage is irreversible in late stage hydrocephalus with cellular framework loss but may not occur in early stages, whether by exclusion of fluid or otherwise. Further studies that are needed to explain the development of hydrocephalus are outlined.

## Introduction

This is the fifth in a series of in-depth reviews [1–4] on the extracellular fluids of the central nervous system. The first three covered the principles and mechanisms of the formation, composition, circulation and outflow of these fluids under “normal” conditions; the fourth considered the mechanisms of extravascular solute fluxes into and out of the brain parenchyma. This present review updates and extends coverage of these aspects to include normal regulation of extracellular fluid volumes and of the stresses and pressures within the brain. It also summarizes and interprets what is known about the physical and (patho)physiological changes occurring in intracranial hypertension, ventriculomegaly, and hydrocephalus.

## Normal conditions

### The extracellular fluids of the brain

**Fig. 1:**
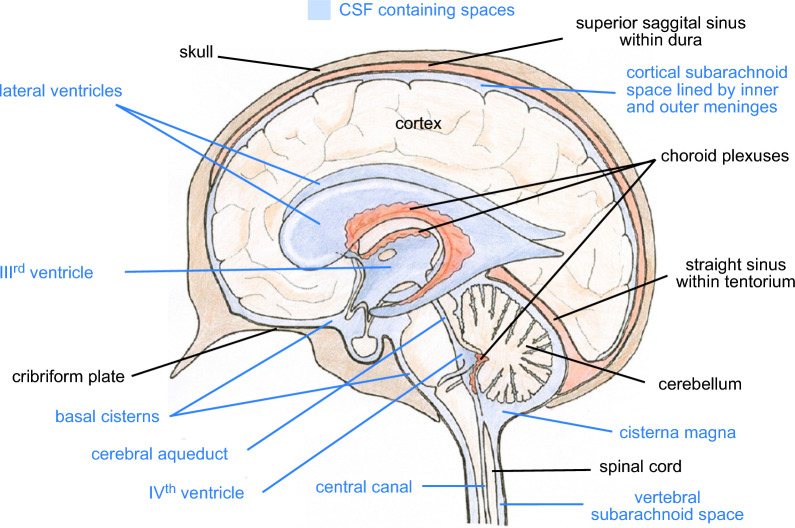
Sagittal section of the brain showing key structures containing cerebrospinal fluid (CSF). The ventricles and central canal of the spinal cord are lined by an ependymal layer. The subarachnoid spaces, including the cisterns at the base of the brain, are lined on the brain parenchymal surface by the glia limitans, comprised of several layers of astrocyte endfeet, and by the inner meninges, a thin layer called the pia. The outer linings to the subarachnoid spaces and cisterns, the outer meninges, are called the arachnoid and the dura. These layers separate the subarachnoid spaces from the skull and vertebrae. The arachnoid is a thin layer thought to be impermeable except where penetrated by nerve roots or blood vessels or specialised structures, the arachnoid villi and granulations. The dura is thicker with considerable mechanical strength. The cortical subarachnoid space surrounds the cortex. The superior sagittal sinus, the largest of the venous sinuses, is shown going over the top of the cortex. It is a tube confined within the dura near the sagittal (midline) plane. The straight sinus is shown above and posterior to the cerebellum. This is contained within the tentorium, a fold of the dura said to provide a tent shaped cover over much of the cerebellum. The figure is derived and relabelled with permission from [5]

**Fig. 2:**
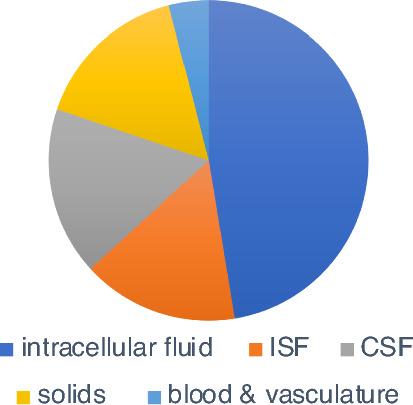
Pie chart illustrating the relative volumes occupied by the various components within the cranium. The total intracranial volume of a typical adult human is about 1470 mL [6]. It contains intracellular fluid (dark blue), interstitial fluid (ISF, orange), cerebrospinal fluid (CSF, grey), solids (yellow) and blood (light blue). Blood occupies approximately 60 mL (4% of the total). CSF volume within the skull is around 250 mL of which roughly 33 mL is in the ventricles. The cells and ISF in the parenchyma together occupy 1160 mL of which ~ 20% is ISF [7], ~ 20% solids [8] and ~ 60% intracellular fluid. References are given in footnotes 1 and 2

The extracellular fluids within the brain may be sub-divided into blood plasma within the blood vessels, cerebrospinal fluid (CSF) that fills the ventricles, cisterns and sub-arachnoid spaces and interstitial fluid (ISF) that fills the gaps between the cells in the brain parenchyma (see Fig. 1). The relative volumes occupied by these various fluids, by intracellular fluid within the cells and by the solid elements of the brain are indicated in Fig. 2,^1^^,^^2^. There is a continuous interchange of water and solutes between the various extracellular fluids.

The gaps between the cells in the parenchyma are narrow, typically 20 nm to 60 nm [9, 10] but the surface area of the gaps is large and the volume of the gaps is typically 20% of that of the parenchyma [10]. Narrow gaps minimize distances for diffusion of substances like neurotransmitters between cells. However, it is also important that the volume of ISF be large enough to accommodate the K^+^ that emerges from neurons during action potentials without significantly altering [K^+^]_ISF_ since this is an important factor in determining neural excitability.

The sizes of the spaces filled with CSF at normal intracranial pressure (*ICP*) (see Sect. 5.3) are determined by whatever processes determine the normal structure of the brain (for some discussion see [11]). The ventricles originate as parts of the lumen of the neural tube which, while still filled with amniotic fluid, becomes sealed at both ends. This neural tube lumen eventually becomes the ventricles, the cerebral aqueduct, and the central canal of the spinal cord. Around the same time that the choroid plexuses form and begin to secrete CSF, openings appear in the wall of the IVth ventricle. These allow fluid to flow out into the forming subarachnoid spaces [12]. At some stage, maintenance of a raised ventricular pressure is an important part of normal development [13, 14], but in the adult, as will be discussed later, there is normally only an imperceptible gradient of pressure between the ventricles and the subarachnoid spaces. However, even in the adult, the structure and volumes of the CSF containing spaces are not fixed and immutable. Anything that changes the dimensions of the spaces, e. g. loss of brain cells, will affect the volume of CSF at normal *ICP*. Similarly changes in the rate of production of CSF or in the resistances to its flow within or out of the brain will via changes in CSF volumes alter the dimensions of the CSF-containing spaces. It is sometimes unclear what is cause and what is effect.

The principal functions of CSF in the adult are:

- To provide partial buoyancy for the brain such that it is not excessively deformed by pressing against the meninges and skull;
- To move easily about the brain thus reducing pressure gradients [15];
- To move between brain and vertebral column to facilitate changes in cranial blood volume during the cardiac and respiratory cycles;
- To remove, non-selectively, a variety of waste substances from the brain via fluid flow [3, 4, 16–18]; and
- To provide a means for transport of substances including vitamins and hormones throughout the brain as a whole, sometimes called volume transmission [19, 20].

There is exchange of materials between CSF and ISF across the ependymal lining of the ventricles and across the pial layer that separates the parenchyma from the subarachnoid spaces. CSF-ISF exchange is augmented by convective movements of ISF through white matter into the ventricles and of ISF and/or CSF through perivascular spaces associated with blood vessels coursing between the parenchyma and the subarachnoid spaces.

CSF is secreted by the choroid plexuses (see Fig. 1) into the brain ventricles. From there it flows to sites of outflow to lymph or blood.^3^ There is also extensive and rapid exchange of water and some solutes across the brain microvessels that provide the blood–brain barrier separating ISF and blood. This fluid may mix with and contribute to CSF.

## Fluid movement into, through, and out of the brain

### Entry via the choroid plexuses

A large proportion of net fluid entry into the brain occurs as active secretion of CSF by the choroid plexuses^4^, one located in each ventricle [14, 21] (and [22] for a recent review on measurement of CSF production). The proportion of the total CSF secretion that occurs into each ventricle may vary with species with the choroid plexuses in the lateral ventricles becoming more important in those species with larger cerebral cortices. Thus in cats, dogs, rabbits and even rhesus monkeys [23, 24] the choroid plexus in the IVth ventricle may secrete more CSF than the other choroid plexuses combined (see discussions in chapter 6 of [7] and in [25]). However, in man, the weight of the IVth ventricular choroid plexus has been reported as only about 15% of the total weight of the plexuses [26] which may suggest that in humans the large majority of CSF is produced within the lateral and III^rd^ ventricles. This is supported by MRI measurements that have found net flow through the cerebral aqueduct similar to estimates of the total rate of CSF production.^5^

The choroid plexus is an excellent example of a secretory epithelium with leaky tight junctions^6^ optimized for a high rate of fluid transfer. Evidence supporting this statement together with the current state of knowledge about the detailed mechanisms involved have been presented and discussed elsewhere [2, 7, 21, 27–31]. There is general agreement that, as expected for an active process, CSF production is relatively insensitive to *ICP* and to the hydrostatic pressure difference across the choroid plexuses [7, 32, 33]. However, it is almost inconceivable that pressure differences would have no effect at all on an epithelium with leaky tight junctions. Flow via the paracellular route between the epithelial cells is a passive process and thus should be pressure sensitive. The evidence for a small effect of pressure has been summarized by Welch [33].

### Entry (and exit) via the blood–brain barrier

The endothelial cells lining the brain vasculature that form the blood–brain barrier provide a large surface area for exchange of fluid and solutes between ISF and blood. The blood–brain barrier is the site of major and rapid influxes and effluxes of water^7^, O_2_, CO_2_ and glucose into and out of the parenchyma. However, exchanges of Na^+^ and Cl^−^ across this barrier are only comparable in size to exchanges of these ions across the considerably longer distances between most of ISF and CSF (see [3] for discussion and references). This is possible because the endothelial cells of the blood–brain barrier differ from those lining the peripheral capillaries and venules. At the blood–brain barrier but not in peripheral vessels the endothelial cells are linked by non-leaky, tight junctions that greatly reduce paracellular transfers including those of Na^+^ and Cl^−^.

The permeability to water and solutes of the layer of glial endfeet surrounding cerebral blood microvessels is normally higher than that of the vascular endothelial layer itself. This is largely a result of the gaps between the endfeet. Furthermore, the presence of aquaporin 4 (AQP4) water channels in the endfoot membrane facing the microvessels increases the water permeability of the endfoot layer.^8^ The presence of this layer has largely been ignored in discussions of the normal transport functions of the blood–brain barrier. Water fluxes across the endfoot layer do need to be considered in discussions of the extent and time course of oedema [34].

#### Net flux of water across the blood–brain barrier

Water easily crosses the blood–brain barrier but nevertheless hydrostatic pressure driven net water transfer across the barrier is much less than the net water transfer that is part of CSF production by the choroid plexuses. Even though the blood–brain barrier is much less permeable to water than peripheral capillaries, water can still cross easily as shown by: rapid equilibration of labelled water; the net flows that can be produced by imposed osmotic gradients; and changes in water relaxation times seen using NMR [35–41]). Half-times for the approach towards osmotic equilibration by water movement across the blood–brain barrier (and the glial endfoot layer) are estimated to be about 10–15 min [39]. Simple diffusion of water across the lipid membranes and cytoplasm of the endothelial cells may account for the water permeability because such diffusion over short distances is relatively rapid and the water solubility in the membrane is adequate as water molecules are so small (see [42] and section 4.3.6 in [2]). There appear to be very few if any aquaporins in the endothelial cell membranes [43] but routes for transfer of water may be provided by various other proteins including the sugar transporter GLUT1 (see [2] for further discussion).

In peripheral capillaries, both water and small solutes, e.g. NaCl, are highly permeable but large molecules, the colloids primarily serum albumin, are not. As Starling described in 1896 [44], in peripheral capillaries, fluid composed of water and small solutes easily moves down the combined gradient of hydrostatic and *colloid* osmotic pressures, a process now called the Starling mechanism (see Appendix A).

By contrast, the Starling mechanism does not describe the movements of water and solutes across the blood–brain barrier. Because the permeability of the barrier to NaCl is very low and its concentration on both sides of the barrier is relatively high, NaCl is much more important than the colloids in producing the osmotic pressure gradient affecting movement of water across the barrier [35, 45–48] (see Appendix A). Fluid, mainly water, will move down the combined gradient of hydrostatic pressure and *total* not just *colloid* osmotic pressure. This process can be described as filtration regardless of whether the net flux of water occurs by diffusion or flow (see Appendix A). Importantly, because NaCl permeability is very low, hydrostatic pressure driven fluid movements across the blood–brain barrier are small compared to the rates of CSF production and outflow.

In order to transfer fluid across the blood–brain barrier at a comparable rate to that at the choroid plexuses, it is necessary for the barrier to secrete or actively absorb the fluid. (For further discussion and references see Appendix A, sections 2.5.1 and 2.7 and footnote in [1] and section 4.2 and footnote 11 in [2]). It is possible, but not yet proven, that fluid secretion from blood to parenchyma does occur with the rate for the whole brain comparable to, but probably smaller than, the combined rates of secretion of fluid by the choroid plexuses (see section 4.1 in [2] and section 5.5.2 in [4]).

The water permeability of the blood–brain barrier is high enough that, at least at sites far enough removed from the choroid plexuses, ISF, CSF, and plasma have nearly the same osmolality.^9^ Just how close the osmolalities of CSF and ISF are to that of plasma is considered in Appendix B.

On present evidence, active net absorption of ISF across the blood–brain barrier is not a major contributor to the normal outflow of CSF from the CNS. It may, however, be sufficient to account for part or all of the much smaller outflow of fluid from the parenchyma involved in the slow resolution of oedema (see [34]).

### Fluid movements within the brain

The way in which CSF is thought to flow through and around the brain has been elucidated using a number of different techniques. These include investigations using injected markers that can be detected by their radioactive emissions as in cisternography or by various types of microscopy or by magnetic resonance imaging (MRI). Early studies using radioisotopes revealed a progression of fluid from the ventricles into subarachnoid spaces with eventual removal from the brain. 24 h after addition, radiotracer was still present on the dorsal surface of the brain but had been cleared from other sites [49–52]. Similar results but with shorter retention times were obtained in rats [53, 54]. More recently, similar time courses have been seen with MRI using gadolinium as a marker for CSF in guinea-pigs [55] and humans [56, 57]. These are the results to be expected where there is less flow through the cortical subarachnoid spaces than through the ventral spaces. This and other proposed explanations for higher dorsal than ventral concentrations at long times after cisternal injection are considered in Appendix C.

**Fig. 3:**
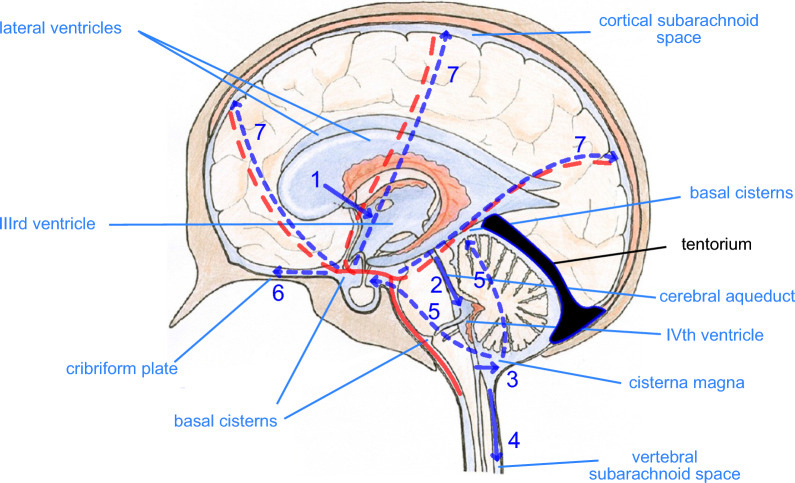
Major routes of net flow of CSF. CSF is produced by choroid plexuses in the lateral, III^rd^ and IV^th^ ventricles. 1) Flow from lateral ventricles into III^rd^ ventricle. 2) Flow through the cerebral aqueduct from the III^rd^ to the IV^th^ ventricle. 3) Flow from the IV^th^ ventricle to the cisterna magna, one of the basal cisterns. From the cisterna magna there is 4) flow into the vertebral subarachnoid space and 5) flow into the other basal cisterns. In many species there is 6) flow from the basal cisterns via the extracellular space of the olfactory nerve to the cribriform plate. 7) Flow from the basal cisterns to cortical subarachnoid spaces occurs primarily via perivascular spaces of cerebral blood vessels anterior to the brain stem. These blood vessels run and branch within the subarachnoid space along the brain surface prior to turning and penetrating into the cortex. Over most of its extent the tentorium is a thin fold of dura separating the cerebellum and cerebrum, but it is much thicker near the sagittal midplane where it encloses the straight sinus (see Fig. 1). It is emphasized here in black because it provides both a mechanical support for the cortex and a barrier to flow of CSF. The solid red line represents arterial inflow to the brain, the dashed red lines indicate schematically the courses of the anterior, middle and posterior cerebral arteries within the subarachnoid spaces at the surfaces of the cortex. Blue arrows indicate CSF flows, those that are dashed being not necessarily in the plane of the figure. Base figure reproduced with permission from [5]

Our current understanding of the principal routes of net CSF flow is summarized in Fig. 3 (compare Fig. 1 in Hammock et al. (1974) [58]). In broad outline, a large proportion of CSF is produced by the choroid plexuses in the lateral and IIIrd ventricles. From the IIIrd ventricle CSF flows through the cerebral aqueduct into the IVth ventricle, crossing from the forebrain (supra-tentorial) to the hindbrain (infra-tentorial). More CSF is added, produced by the choroid plexus in the IVth ventricle and the total CSF then flows into the cisterna magna located behind the brain stem and below the cerebellum. The cisterna magna is one of the enlargements, the basal cisternae, that are found surrounding the brain stem and extending across the base of the cranial cavity [59–61]. Normally there is free flow of CSF from the cisterna magna into the vertebral subarachnoid space and through the basal cisternae. In rodents and sheep there is also free flow of CSF to the olfactory cistern and thence to the olfactory nerve and cribriform plate. Flow to the cribriform plate may be less prominent in humans ([62–65]).

Flow from the subarachnoid spaces below the level of the tentorium up to the cortical subarachnoid spaces is usually less prominent than the flows towards the cribriform plate and vertebral subarachnoid space and occurs primarily through the perivascular spaces of the cerebral blood vessels running within the cortical subarachnoid space ([62, 63, 66–69]) (reviewed in section 5.1 in [4]) (see note added in proof). These vessels spread over the cortical surface having coursed through the basal cisterns and having crossed into the forebrain anterior to the brain stem. Transfer posterior to the brain stem is restricted to regions close to the stem by the presence of the tentorium, a large thin fold of dura that separates the cerebrum from the cerebellum [70, 71].

#### Pulsatile flow of CSF

MRI studies show clearly that net flows of CSF are superimposed on much larger back and forth movements driven by hydrostatic pressure changes produced by changes in blood volume throughout the parenchyma. These changes occur in time with the cardiac and respiratory cycles and result from changes in blood volume in the microvasculature of the parenchyma [72–83]. During systole, more blood enters the cranium than leaves and this drives CSF out of the cranium into the vertebral subarachnoid spaces. Most of the outward flow originates from the cranial subarachnoid spaces, the rest from the ventricles. During diastole, the flows are reversed.

The flows into and out of the vertebral subarachnoid spaces through the foramen magnum are much larger than those through the aqueduct but those through the aqueduct have been measured more often (see Table 12.1 and Figs. 12.4 & 12.10 in [82, 84, 85]. The amounts of CSF transferred in the two directions through the aqueduct are almost the same with the average called the aqueductal stroke volume (or aqueductal CSF stroke volume, ACSV [86]). Superimposed on the cardiac-driven changes are less frequent but sometimes larger changes driven by the respiratory cycle [87, 88]). The ability of CSF to move through the ventricles and subarachnoid spaces of the brain and vertebral column is important to allow changes in cranial blood volume without large local changes in pressure. It has been suggested by some that failure of this mechanism can play an important part in the development of hydrocephalus (see Sect. 9.2.3).^10^

Normally (in the absence of hydrocephalus), marker substances added to CSF in the lateral ventricles pass through the III^rd^ and IV^th^ ventricles, so called anterograde motion. There is very little if any penetration into the III^rd^ and lateral ventricles of markers added via the lumbar sac or cisterna magna [89–91]. Because the IlIrd and IVth ventricles and the foramina between the IVth ventricle and the cisterna magna all allow movements in both directions, this lack of significant retrograde transfer implies that the stroke volume in the aqueduct must be substantially less than the volume of the aqueduct.

#### Fluxes and flows between the CSF-containing spaces and the parenchyma

**Fig. 4:**
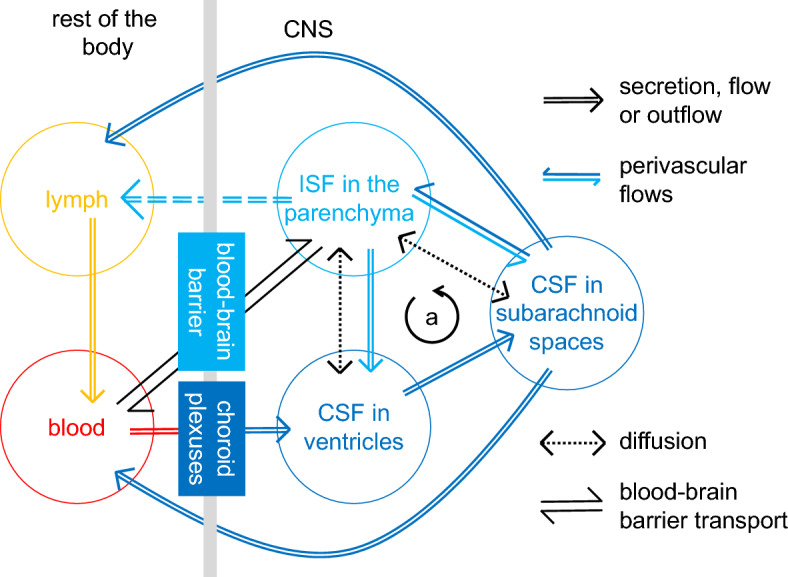
Formation and circulation of cerebrospinal fluid (CSF) and interstitial fluid (ISF). CSF is formed from blood by secretion across the choroid plexuses into the ventricles. There may also be a contribution to CSF from ISF secreted by the blood–brain barrier. Outflow of CSF from the brain occurs from the subarachnoid spaces to lymph and blood (solid dark blue lines). There may also be outflow of ISF from the brain to lymphatics via pathways (possibly a subset of perivascular spaces) that bypass the CSF (dashed light blue lines). There is diffusional exchange of solutes between ISF and CSF across the outer pial lining separating the parenchyma from the subarachnoid spaces and across the ependyma that lines the ventricles (dotted black lines). Convective transfer of solutes between the subarachnoid spaces and the parenchyma occurs along perivascular pathways. This transfer may be mediated by net flows of fluid along separate perivascular pathways or they may be via movements through a common pathway. The relative sizes of the inward and outward fluxes of solutes by these pathways are not known. There may also be a net outward flow of ISF from white matter into CSF within the ventricles. As indicated by the circular arrow labelled a) there may be a recirculation of fluid from ventricles to subarachnoid spaces to ISF and back to CSF in the ventricles (compare [100]). This recirculation complicates determinations of the rate of CSF production by the choroid plexuses. There may also be a recirculation of CSF into ISF and back to CSF by perivascular routes. Alternatively or in addition, there may be net flow of CSF into ISF and then into lymph also via perivascular pathways

There is good evidence for fluxes of solutes and probably also flows of fluid between CSF and ISF (reviewed in [4, 92]) as indicated in Fig. 4. On present evidence, the most likely scenario is that there is a component of net CSF flow into the parenchyma along extramural arterial perivascular spaces as proposed by Rennels et al. [93] and Iliff et al. ([94] which may account for inward movement of solutes like horseradish peroxidase [93] and amyloid-β [95] when they are added to CSF. There is a net ISF flow out of the parenchyma via "preferred" or "preferential" routes which may include routes leading to CSF in the ventricles and subarachnoid spaces and/or to lymphatics in the meninges [92, 96–98]. Perivascular spaces and other preferred routes of extravascular movements of solutes and fluid are often called glymphatics [94]. The relative importance of perivenous spaces, intra- or extramural periarterial spaces and white matter tracts as routes of efflux of solutes and outflow of fluid is still under consideration [4, 92, 98, 99].

The net flows in the parenchymal perivascular spaces are superimposed on other forms of convection and it is this combination of forms of convection that accounts for both the delivery and the removal of solutes along arterioles and venules (compare Fig. 5 in [1]). The driving force for convection along arteries within the parenchyma may well involve vasomotion, i.e. local smooth muscle contractions in the blood vessel walls [101–105] and/or the dilations and contractions of the arteries that occur as part of neurovascular coupling [106–108]. Convection along perivascular routes and white matter tracts and simple diffusion through the interstitial spaces of grey matter probably account for the effluxes from the parenchyma of solutes that can neither be metabolized within the brain nor cross the blood–brain barrier^11^ (for further discussion see section 4.3.4 in [1], [109] and section 3.2 in [3]). Of particular interest here, *net* transport of Na^+^ and Cl^−^ appears to occur at similar rates across the blood–brain barrier and via extravascular pathways (see section 4.3.2 in [2] and section 5.6 in [3]) and thus both of these routes are likely to be important in the development and resolution of oedema [34].

### Fluid outflow from the CNS

**Fig. 5:**
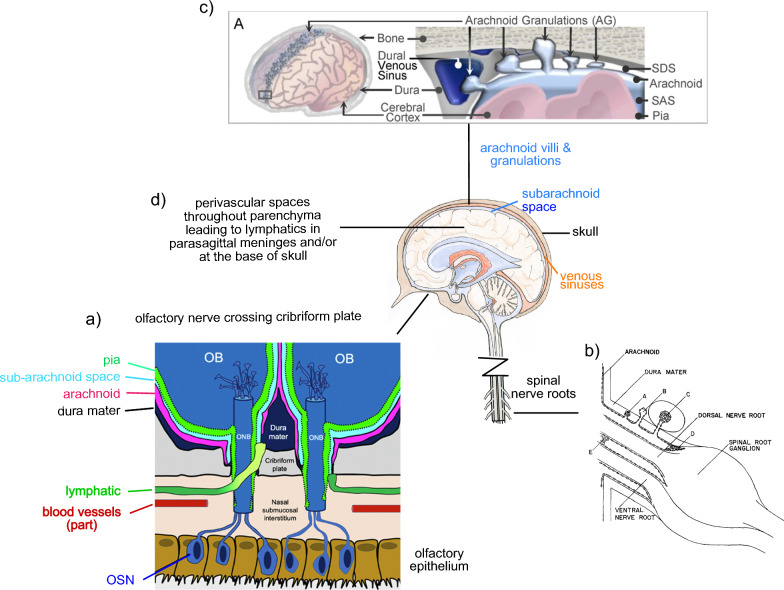
Schematic diagrams of various routes of outflow of CSF from the central nervous system, listed here in a presumed order of importance (see text). **a** Perineural pathways, prominently along the olfactory nerve leading via the cribriform plate to lymphatics in the nasal submucosa. In this pathway large solutes enter lymphatics, thought in mice to be via direct connection between the perineural (subarachnoid) space and the lymphatics (OB, olfactory bulb; ONB, olfactory nerve bundle; OSN, olfactory sensory neuron) [115]. In older literature this type of route for outflow was called closed-cuff [116] because it is "closed" to transfer of large solutes between the subarachnoid space and the nasal interstitium. Possible differences between species are discussed in the text of this section. **b** Arachnoid villi like structures at the roots of spinal nerves leading to peripheral interstitial fluid and/or lymphatics. A to E refer to different positions and possibly different functions for these structures [117]. **c** Cranial arachnoid villi (granulations are large villi) leading either to the sagittal or transverse sinuses in the dura or to peripheral interstitial spaces in the meninges [112]. SDS, subdural space; SAS, subarachnoid space. **d** Perivascular spaces and possibly cranial arachnoid villi leading to lymphatics in the cranial meninges located close to the dural sinuses or at the base of the skull. Subfigures **a**, and **b** are reproduced with permission from the sources indicated, subfigure c from [112] is available under a Creative Commons License (Attribution 4.0 International, as described at https://creativecommons.org/licenses/by/4.0/)

The nature of the routes of fluid outflow from the CNS have been reviewed recently [110, 111]. As indicated in Fig. 5 there are four principal pathways that may be important^12^:

- the extracellular spaces contained within the sheathes of cranial nerves, especially the olfactory nerve leading via the cribriform plate to the nasal mucosa;^13^
- arachnoid-villi-like structures located at the roots of spinal nerves leading to peripheral interstitial fluid and/or lymphatics;
- cranial arachnoid villi (which include arachnoid granulations) leading either to venous sinuses or to peripheral interstitial fluid and thence to lymph [112–114]; and
- perivascular spaces leading to lymphatics in the cranial meninges or, especially if there are intramural spaces within the walls of blood vessels (the intramural periarterial drainage (iPAD) hypothesis^14^), at the base of the skull.

#### Cranial perineural routes

**Fig. 6:**
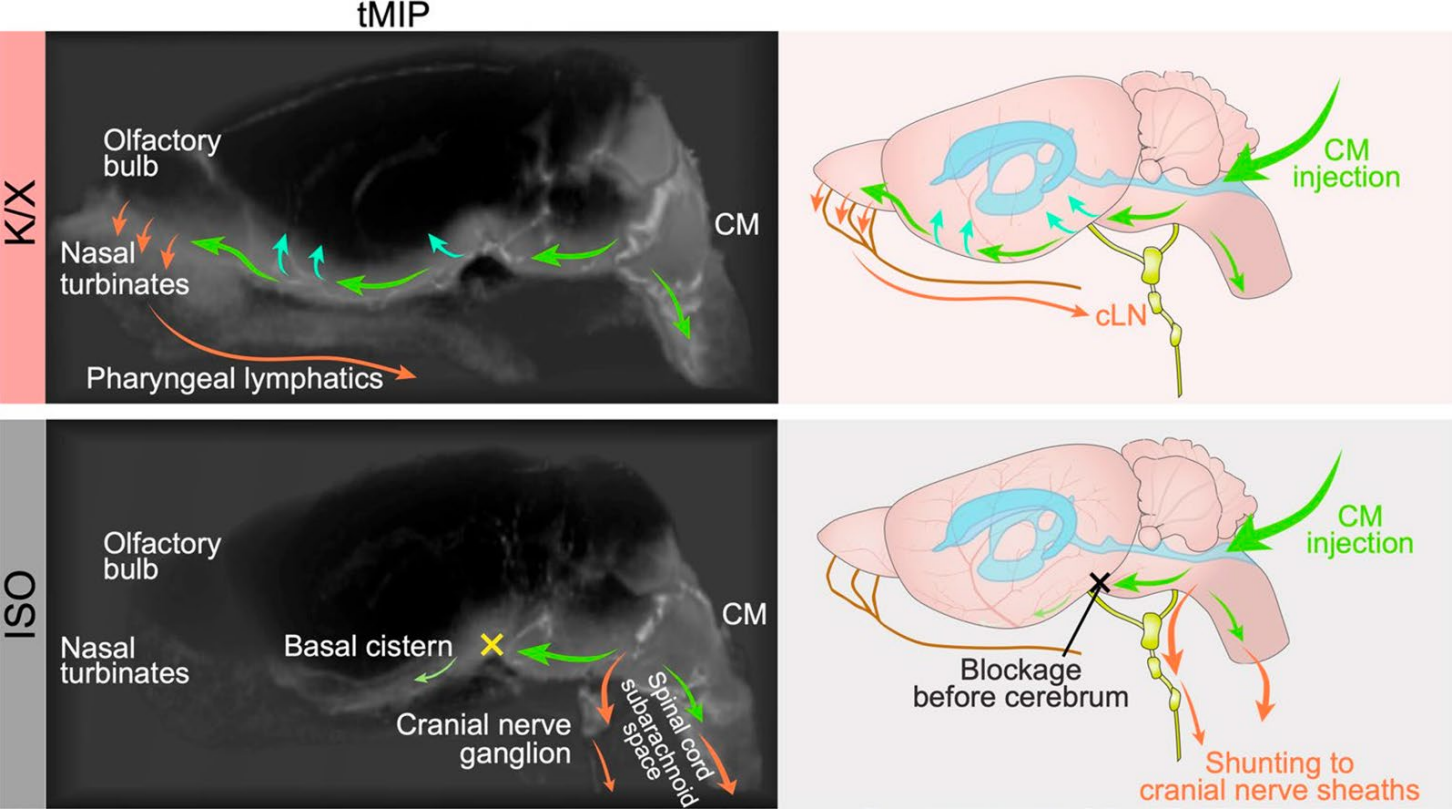
Principal CSF flow routes under ketamine/xylazine (K/X) and isoflurane (ISO) anaesthesia in mice. Time maximum intensity projection (tMIP) on left and schematic diagram on right. Gadobutrol injected into the cisterna magna (CM) was followed by magnetic resonance imaging. Green arrows indicate routes of CSF flow within the brain and upper spinal cord, red arrows routes of CSF outflow. Under ketamine/xylazine anaesthesia the gadobutrol spread primarily through the basal cisterns to the olfactory nerve, across the cribriform plate and into the nasal turbinates and pharnygeal lymphatic vessels. It could also be seen to enter the vertebral subarachnoid spaces and to spread over the cortical surface close to the middle cerebral artery. By contrast under isoflurane anaesthesia the gadobutrol outflow was almost entirely via the sheathes of cranial nerves originating in the brain stem or routes leading out of the vertebral subarachnoid spaces and the spinal nerve roots. cLN, cervical lymph node. Reproduced with permission from [153]

The importance of cranial perineural routes, prominently the olfactory nerve crossing the cribriform plate (see Figs. 1, 6), is now well established in cats, rats, mice, rabbits, sheep and non-human primates [54, 111, 113, 118–138]. In humans the importance of the route via the cribriform plate is less clear. Imaging of 18F-THK5117, an intravenously administered tau pathology tracer, showed prominent delivery of the tracer to the nasal turbinates [65, 139]. By contrast, gadobutrol given intrathecally did not accumulate within the nasal mucosa even though it could be seen penetrating to below the cribriform plate [64]. In a separate study, gadobutrol was seen to have accumulated adjacent to the cribriform plate [140]. It is unclear how to reconcile these results [64, 65, 140]. Melin et al.[64] carefully avoided reaching firm conclusions but, based on their results, favored the view that the cribriform plate is a minor route for elimination in humans. Alternatively, their data may favor either (i) a closed-cuff model for the connections between the perineural routes through the plate and lymphatics [111, 115] (for discussion of open versus closed cuff models see [110]) or (ii) sufficiently fast removal of gadobutrol in lymphatic outflow or venous outflow (see [141] but also [136]) that concentrations in the nasal mucosa remain low. Mehta et al. [65] contains extensive discussion of the importance of the nasal route in humans.

Intranasal drug administration has been demonstrated in experimental studies as a route for direct delivery to the brain [142]. This result would be difficult to understand if there were no CSF outflow via this route. At present all that can be concluded is that, while unlikely to be negligible, the relative importance of CSF outflow via the cribriform plate in humans is still not known (for consideration of further indirect evidence see Sects. 3.4.3 and 7.1).^15^

#### Vertebral routes

Outflow from the vertebral subarachnoid space primarily to lymphatics is likely to be substantial (at least 20% of the total from the CNS) [141, 143–153] especially in upright primates [154] (for further discussion see [110, 111]) (see note added in proof).

#### Cranial arachnoid villi

The importance of CSF outflow via cranial arachnoid villi to venous sinuses is presently controversial despite this having been accepted as the principal route from early in the twentieth century. The flow patterns of CSF measured in humans [56, 57] are consistent with the outflow of a major fraction of the CSF via cranial nerves and spinal nerve roots with markers reaching and leaving the dorsal subarachnoid spaces relatively slowly [4, 146]. Efflux of substances from CSF directly to the sinuses has not been observed (but would be difficult to measure [110, 111, 155]). If it does occur, it may be too slow relative to blood flow through the sinuses to be measured. Alternatively, it may be that solutes leaving via arachnoid villi are delivered to the dural extracellular space and lymphatics rather than directly to venous blood [112–114, 156, 157].

There is, however, indirect evidence for outflow to venous sinuses. Firstly, following production of a silicone plug in the basal cisterns of dogs, transfer to plasma after suboccipital injection of labelled-albumin was delayed and slowed with the increase in the amount of albumin being coincident with its delayed arrival in the dorsal subarachnoid spaces [146].

Secondly, venous obstruction or increased right atrial pressure increase venous sinus pressures which should reduce any outflow via arachnoid villi leading to the sinus and thus be associated with increased *ICP* (see Sect. 3). Furthermore, stenting to relieve venous obstruction and lower pressure in the sinuses, reduces raised *ICP* towards normal [158–160]. However, the observation of raised sinus pressure in connection with raised *ICP* does not provide an irrefutable argument either that the increased sinus pressure is the cause of the raised *ICP* or that outflow of CSF occurs directly to the sinus. Increased *ICP* will necessarily compress the sinuses and this is held to be particularly marked in the sinus regions nearest their sites of outflow [161, 162] which would lead to upstream increases in sinus pressure. Similarly raised sinus pressure will be associated with raised pressure in the surrounding tissue which may affect routes of outflow other than directly to the sinuses (for further discussion see Sect. 7.1).

#### Perivascular routes and lymphatics in the cranial meninges or/and at the base of the skull

Evidence demonstrating that there is efflux of substances to lymphatics in the cranial meninges or at the base of the skull is convincing [112, 163–168], but it is not clear what fraction of fluid outflow occurs by these routes [111, 168]. Neither *ICP* nor brain fluid volumes are changed when meningeal lymphatics are ablated [168], but this could mean either that these lymphatics are of minor importance in fluid outflow or that when the lymphatics have been ablated there are compensating changes in other outflow pathways and/or in CSF production rate (compare [169]). Outflow from the parenchyma direct to lymphatics may be of particular importance in providing a route for outflow of ISF that does not entail mixing with CSF in the ventricles or subarachnoid spaces as will be discussed in Sects. 5.2.1 and 9.2.2.3.1 (see also section 5.6 in [4]).

The relative importance of the outflow routes described above varies with circumstance. In this regard, Stanton et al. [153] found that in mice under ketamine/xylazine anaesthesia the outflow route was primarily via the cribriform plate but under isoflurane anaesthesia the outflow was primarily via cranial nerves originating in the brain stem and to a lesser extent via spinal routes (see Fig. 6). Thus the principal sites of outflow are different, but in neither case is outflow prominent from the cortical subarachnoid space. Given the importance that changes in outflow route must have in hydrocephalus, further studies to determine routes of outflow in humans are clearly needed (compare [64]). Regardless of the specific routes for outflow, the total rate of outflow increases markedly with intracranial pressure (see Sect. 5.3).

## Regulation of Na^+^ and extracellular fluid volume

Salts of Na^+^ are by far the principal contributors to the osmolality of the extracellular fluids in the brain as in the rest of the body, i.e. qualitatively osmolality 2 × [Na^+^]. Because water can enter and leave the brain relatively rapidly, the osmolality of brain fluids is close to that of plasma (see Appendix B). With osmolality controlled, the distribution of the extracellular fluid volume between the brain and the rest of the body is regulated by the transport of Na^+^ salts into and out of the brain.

The major routes of these transfers are via CSF production, outflow of CSF (and possibly ISF) to lymph and blood, and net flux across the blood–brain barrier. Transport of Na^+^ across the blood–brain barrier has been considered in detail in sections 4.3.3 to 4.3.5 in [2]. There is evidence for both large, nearly equal passive fluxes in both directions and active transport resulting in a net flux from blood to brain. In contrast with the net flux, radiotracer fluxes of Na^+^ across the blood–brain barrier are unsaturable and unaffected by transport inhibitors. The mechanism for the large undirectional passive fluxes is likely to be simple electrodiffusion through the tight junctions.

The active net flux is necessarily transcellular. The endothelial cells of the blood–brain barrier have Na^+^-pumps in their abluminal membranes that mediate net flux of Na^+^ from the endothelial cells to ISF and net flux of K^+^ in the other direction. The ATP needed to drive these pumps is produced by the high numbers of mitochondria typically present in the brain microvascular endothelial cells. It is plausible that together with other transporters, notably the Na^+^, K^+^, 2 Cl^−^ cotransporter in the luminal membrane, the pumps mediate a net flux of NaCl from plasma into ISF with water following by some combination of osmosis and coupled-transport. If it occurs, this would amount to a secretion of ISF by the endothelium into the parenchyma.

Under normal conditions, because the Na^+^ concentrations and electrical potential are almost the same on the two sides of the barrier, the net flux via the passive mechanism is likely to be small. All available data are consistent with [2]

$$\begin{array}{lllll} {\begin{aligned}&{\text{passive, paracellular}} \\ &{\text{unidirectional fluxes}}\end{aligned}} & >> & {\begin{aligned}&\text{transcellular net} \\ &\text{flux}\end{aligned}} & > & {\text{paracellular net flux}}\end{array}$$

which allows the net flux across the blood–brain barrier to be affected by inhibitors of transcellular processes while the measured unidirectional fluxes are not.

The passive net flux is predicted to become very important in regions where there are substantial differences in Na^+^ concentrations on the two sides of the blood–brain barrier as occurs in focal ischaemic oedema [34].

## Volumes, stresses and pressures within the cranium

### Volumes of CSF, ISF and the parenchyma

Very little is known about the factors determining the normal volumes of the spaces occupied by CSF. For further discussion of the development of these spaces see e.g. [11]). The volume of ISF within any part of the parenchyma is determined by the dimensions of the spaces between the cells.

#### The parenchyma as a framework of cells with the gaps between filled by ISF

The brain parenchyma can be viewed as a porous solid consisting of a framework of interlinked cells with the spaces between them filled with ISF. There is also a network of blood vessels. ISF and blood are liquids that can flow. In contrast, the framework of cells is an easily deformable solid. However, to a good approximation neither can be compressed, i.e. they have constant density. Thus the volume of the parenchyma can be changed only by changes in the amounts of substances present, i.e. inflow or outflow of fluids or, in the longer term, loss or gain of cells or other solid components.^16^

### Stresses and pressures

#### Defining terms: stress on a solid; pressure on a liquid

The force exerted on or by a solid need not be the same in each direction whereas the force exerted on or by a stationary liquid is the same in every direction. With a solid, force per unit area is called a stress. With a stationary liquid, the stress, the same in all directions, is called a pressure.

**Fig. 7:**
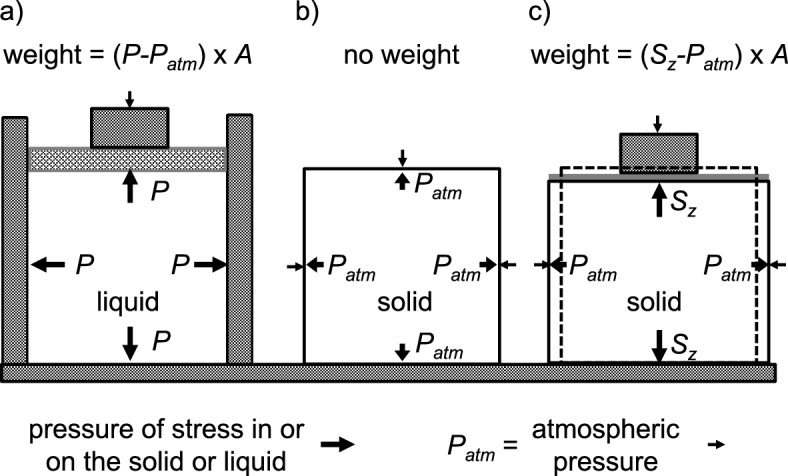
Diagram illustrating how forces act on liquids and on solids i.e. pressure vs stress. **a** The forces exerted on or by a liquid (hatched lines) are the same in every direction and per unit area (*A)* are termed pressure. As shown in **a**, pressure *P* is increased above atmospheric *P*_atm_ by a weight placed on a piston (dark grey stippled rectangle) and by the cylinder walls (grey) containing the liquid. **b** When the force acting on a solid (cross hatched) results solely from atmospheric pressure, it too is the same in every direction. **c** When the same weight as in **a** is placed on the solid, it imposes a stress on the solid that deforms it by decreasing its height (producing a negative vertical strain) and increasing its extent in the two perpendicular horizontal directions (producing positive horizontal strains)

To illustrate how the forces on a solid can differ depending on direction, consider the following. If a cube is compared without (Fig. 7b) and with (Fig. 7c) a weight placed on it, the weight will deform the cube so that it becomes shorter but fatter. Vertically the force is atmospheric pressure, *P*_atm_, times the area of the top surface plus the weight (the vertical stress is *P*_atm_ + weight/area), but horizontally the force is still just the atmospheric pressure (the horizontal stress) times the area of a vertical face. If the solid is incompressible (i.e. it has constant density), the area in the horizontal plane will increase as the vertical height decreases such that the volume remains constant.^17^

#### Stress (S), interstitial fluid pressure (ISFP) and solid tissue stress (STS) in the parenchyma

The parenchyma is essentially a porous solid. It has a solid framework and thus the forces within the parenchyma can vary with direction, i.e. the total force per unit area is a stress. However, within the framework the interstitial spaces are filled with a liquid, ISF, on which the force per unit area, the stress, is the same in all directions and is called a pressure. The overall properties of the parenchyma must be understood in terms of both its solid and liquid components.

Treating the total force per unit area on a porous solid, that is the total stress (*S*), as the sum of two components was first applied to practical problems in 1923 by Karl Terzaghi, who was working on the mechanical properties of soils like clay or sand [170, 171]. In his formulation the first component is set equal to a pressure which is applied to both framework and liquid and the second, now called Terzaghi's effective stress, is the rest of the total stress. The effective stress is applied only to the framework. The difficulty with this formulation is that the effective stress is not equal to the actual stress acting on the framework.^18^

A different separation of the total stress into two components, which avoids the abstract nature of Terzaghi's effective stress, was introduced into engineering in 1941 by Biot [172] and into physiology in the 1960s by Guyton et al. [173]. This formulation makes use of the fraction of a porous solid occupied by the pores (or interstitial spaces), ϕ, to allow separation of the stress into a component due to the pressure within the fluid, called pore pressure or interstitial fluid pressure (ISFP), and a component due to the stress within the framework, which can be called the solid tissue stress (*STS*). It then follows that 19

$$S = \, ISFP \times \varphi + STS \times \, ({1} - \varphi ).$$

Except for a difference in notation, this is the same as Eq. 1 in Guyton et al. [173]. When there is no component of imposed stress that acts solely on the framework, *S* = *STS* = *ISFP,* and the total stress acting on the solid is a pressure.^20^

The most important consequence of this relation for the interpretation of results from experiments on the brain is that placing the solid surface of a pressure transducer against a brain surface measures the total stress perpendicular to the transducer surface, i.e. the perpendicular component of *S*, rather than either the interstitial fluid pressure, *ISFP*, acting on the liquid or the perpendicular component of solid tissue stress, *STS*. But it is gradients of *ISFP* not *S* that will determine whether fluid enters, moves through or leaves the parenchyma. This is a distinction that will be important when considering hydrocephalus.

Unfortunately, *ISFP* in biological tissues cannot be measured directly because there are no pressure transducers small enough that they can be inserted into the interstitial spaces without contact with the framework. However, *ISFP* can be determined by measuring the pressure within free fluid that is allowed to equilibrate with the ISF [173]. Several techniques have been developed to measure ISFP as described in a footnote.^21^

In a liquid there is no framework and the total pressure is the same as the fluid pressure. Within a porous solid there is a framework and the total stress can differ from the fluid pressure. A difference of pressure applied to a liquid will cause it to flow. A difference in total pressure on opposing faces of a porous solid will cause it to move. In the brain any difference in pressure between the ventricles and the subarachnoid spaces will cause CSF to flow and the intervening parenchyma to move until the pressure differences drop to zero or the movement is stopped by contact with other solids.

### Intracranial pressure, *ICP*

The only persistent difference between the total pressures (more precisely stresses) at two locations in the central nervous system are those due to the effect of gravity^22^ unless *both* CSF cannot flow sufficiently rapidly between them *and* the parenchyma is somehow constrained so that the framework of cells cannot be sufficiently deformed to allow it to move down the pressure gradient. If, for instance, total ventricular pressure were increased so that it was greater than total subarachnoid space pressure, the parenchyma would move from the ventricles towards the subarachnoid space. This would increase ventricular volume and decrease subarachnoid space volume. Normally this movement is sufficient to decrease ventricular pressure and/or increase subarachnoid pressure until the pressures are equal. Under normal conditions there are no observable persistent differences in total pressure measured with transducers in the ventricles, subarachnoid spaces and cortical parenchyma in cats [174] or with transducers in the ventricles and cortical subarachnoid spaces of humans [175] (see also [176] for no difference in total pressure between the lateral ventricles and cisterna magna in the face of aqueductal blockage in H-Tx rats). Furthermore, no pressure differences are seen between ventricles, cortical subarachnoid spaces and cortical parenchyma in dogs [177, 178] and humans [179] even in the face of the relatively rapid pressure changes and measurable flows of CSF occurring during the cardiac cycle. The time taken for (total) pressure to equilibrate in the brain is estimated as about 35 ms [180] and the calculated pressure differences during the approach to equilibrium are very small [181]. It is thus normally appropriate to refer to a single but time-varying value of "intracranial pressure", *ICP*, throughout the brain. A more precise definition, the effect of gravity, and caveats and exceptions to the statements made in this paragraph are considered in footnote.^22^

Whereas *ICP* is defined as the pressure measured in a lateral ventricle, in practice it is often measured in the lumbar sac [182] with the effects of gravity minimized by having the subject lie horizontally on their side. Similarly *ICP* monitoring can be carried out with microsensors mounted in a subdural space or in the parenchyma (see e.g. [183, 184]).

*ICP* varies with time, posture (see e.g. [185–187]) and circumstance. Pressure within the brain will change during the cardiac cycle with increased pressure during systole as more blood is pushed into the head and decreased pressure during diastole as blood is removed. Stated values of *ICP* are normally averages over a number of heart beats (and breathing cycles).

*ICP* in healthy people (in a horizontal position) is normally somewhat less than 10 mmHg higher than the ambient air pressure outside of the head (see e.g. [32, 188]. Thus when the skull and dura are opened, CSF emerges and the parenchyma can bulge outwards. Harmful effects of severely raised *ICP* include increased pressure in the optic nerve leading to papilloedema, reduction in cerebral blood flow and herniation of the cortex through the tentorium or of the brain stem or cerebellum through the foramen magnum. Safe limits on *ICP* depend on circumstances. After severe head trauma the prognosis is much better for *ICP* < 20 mmHg than for higher pressures [183, 189, 190].

**Fig. 8:**
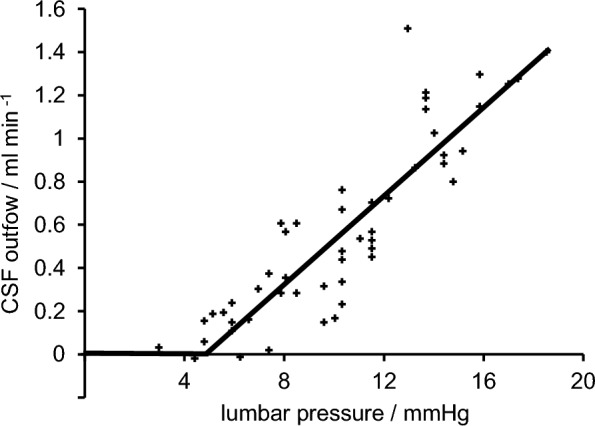
Graph showing the relationship between calculated rates of outflow of CSF by all routes versus hydrostatic pressure in the lumbar sac during ventriculo-lumbar perfusions. Lumbar pressure is likely to be closely similar to that in the cranial subarachnoid spaces and ventricles. Measurements were made in children lying on their sides and given intraventricular fluid perfused at various rates. The curve, two straight lines intersecting at a pressure greater than 0, has been fitted by least-squares. These data can be interpreted as showing that outflow is zero at pressures below a critical value and increases with pressure above this value. Data extracted from Fig. 3 of Cutler et al. (1968) [192]. Comparable data, but over a more extended range, can be seen in [193]

The control of *ICP* is inextricably linked with that of CSF volume. Increases in *ICP* markedly increase the rate of CSF outflow (Fig. 8) [188, 191–193]. This observation does not depend on the specific routes by which the outflow occurs. In the steady-state, the value of *ICP* is that at which CSF outflow balances CSF production (see [7] for extensive discussion).

As noted in Sect. 3.1, CSF production is only weakly dependent on *ICP*. In contrast, fluid outflow by each of the known routes changes whenever there are changes in either *ICP* or the downstream outlet pressure for that route. As a first approximation, assuming the multiple outflow routes can be described as a single equivalent outflow route, this dependence of outflow on pressure can be written as Davson's equation [7, 194–196]

$${\text{outflow }} = \, \left( {ICP - {\text{ outlet pressure}}} \right)/\left( {\text{outflow resistance}} \right) {\text{if }} ICP > {\text{ outlet pressure}}$$

and

$${\text{outflow }} = \, 0 \qquad {\text{if }}ICP \le {\text{ outlet pressure}}.$$

Thus, in the steady-state when CSF production rate is equal to the outflow

$$\left( {\text{outflow resistance}} \right) \, = \, \left( {ICP - {\text{ outlet pressure}}} \right)/\left( {\text{CSF production rate}} \right).$$

Note that this equation defines the resistance regardless of how *ICP* varies with the amount of CSF present, i.e. it does not depend on either the volume of the fluid containing spaces or the compliance of the brain (see next section). Outflow resistance can be calculated from data obtained using constant rate infusions into the subarachnoid spaces. The outlet pressure is estimated as the *ICP* at which outflow can first be detected, or more accurately as the extrapolation to zero flow of a graph of flow versus *ICP*.^23^

Whereas the outflow resistance is relatively straight-forward to calculate (at least in principle), it is important to note that its interpretation in terms of the processes involved is more complex. Much of the complexity arises because the resistance is affected by the relations between *ICP* and the fluid pressures, such as obstructions in the flow pathways between ventricles and outflow sites.^24^

### Two ways to view the relation between *ICP* and CSF volume

The relation between CSF volume and *ICP* can be viewed in two ways. Additions and removals of CSF will produce changes in *ICP*. Thus in a sense the volume determines the pressure. However such changes in volume will not be sustained because increased or decreased outflow will bring the CSF volume and *ICP* back to levels at which CSF production rate and outflow are in balance. Thus, while acutely changing the volume of CSF by injections or withdrawals will produce changes in pressure, in the long-term the need to balance production and outflow of CSF determines both pressure and volume.^25^*ICP* takes on the value that balances input and output and the volume becomes that which produces the *ICP* required.

### Fluid pressures in the brain

The ventricles are filled with a liquid, CSF, in which there is no solid, i.e. no framework, so the total and fluid pressures are equal. The same will be true in subarachnoid spaces only if they are open and stresses borne by the trabeculae that bridge the spaces an be ignored. Cortical subarachnoid spaces may be collapsed except in perivascular regions near blood vessels [66, 67, 197–199]. A pressure transducer inserted into a space smaller than the transducer measures the total pressure at the surface of the solid that is adjacent to the space without revealing the fluid pressure.^26^

Although, as discussed above, the total stress is nearly the same throughout the brain, there must be gradients of fluid pressure responsible for the observed fluid movements (but see footnote 24). The differences of fluid pressure needed in the CSF-containing spaces can be calculated from the observed flows and the measured geometry of the spaces. Even with the varying CSF movements occurring during the cardiac cycle, the pressure differences are below the limits of detection, i.e. at most 1–2 mmHg [80, 178]. However, larger gradients in *ISFP* may be required to drive movements through the parenchyma in hydrocephalus. The existence of such gradients in *ISFP* without gradients in total pressure would imply that there are compensating gradients in the solid tissue stress (see Sect. 5.2.2).

## Conditions where there are pathological changes in brain fluids and pressures

The major processes involved in fluid movements in the brain discussed above are altered in certain pathologies. Intracranial hypertension (Sect. 7), ventriculomegaly (Sect. 8) and hydrocephalus (Sect. 9) are considered in the following sections. Oedema, swelling of brain tissues, is described in [34].

## Intracranial hypertension

Intracranial hypertension is a sustained increase in *ICP*. Regardless of the underlying pathology, raised *ICP* requires that there has been (see Sect. 1.6.3) increased rate of CSF production (rarely [158, 200, 201]), increased resistance to outflow or increased outlet pressure opposing outflow [158, 202, 203] (see Sects. 5.3 and 5.4). Most instances of raised *ICP* result from obstruction of CSF flow by tumors, oedematous tissues, intracranial hematoma or traumatic injury. The raised *ICP* is then often accompanied by hydrocephalus [204] (see Sect. 9) as the distribution of fluid within the brain is also affected.

Intracranial hypertension without obvious obstruction to CSF flow within the brain or changes in ventricular or parenchymal volumes is called idiopathic intracranial hypertension (iIH) [158].^27^^.^Perhaps the most common symptom of iIH is persistent headache. An important sign of seriously raised *ICP* is swelling of the optic disk called papilloedema. Untreated this can lead to permanent blindness.

The incidence of iIH is roughly 0.5—2 per 100,000 per year in the general population but it is much higher, 12–20 per 100,000 per year, in obese women of child-bearing age [205–208].^28^ How female sex and obesity combine to make iIH more likely is still uncertain though changes in venous or right atrial pressures [209–211] (but see also[212]) or altered hormonal levels [200, 203, 208, 213, 214] are under investigation as possible associated risks.

### Increased outlet pressure opposing outflow

Raised pressure in the dural venous sinuses, in regions at the base of the skull or in the nasal mucosa (or the lymphatics draining that region) could all conceivably be causes of raised *ICP* because these are all at the downstream end of potential routes of outflow. Most attention has been paid to pressures in the sagittal and transverse sinuses and changes in the venous system which may affect those pressures [15, 158, 160, 209, 210, 212, 215–217]. Partial stenosis in a sinus, which could explain raised pressure in that sinus and thus raised *ICP*, has often been found in cases of severe iIH [15, 158, 209, 218–222].

Furthermore stenting a stenosis, when present, has been found to be an effective means to reverse the increase in *ICP* [158, 159, 201, 213, 223–226].

Pressure in the venous sinuses is probably larger than normal in all instances of iIH, but this is not in itself a sufficient argument that increased venous sinus pressure is the cause of the increased *ICP*; it may instead be a result (see e.g. [208]). The sinuses are within the skull and thus, even though they are partially protected by the dura, they must still be exposed to raised *ICP* and this will increase the pressures within them [162, 202, 216, 226–234]

Regardless of whether or not stenosis was the initial cause of the increase in *ICP*, it may bring about a positive feedback loop [210, 215, 227, 235]:
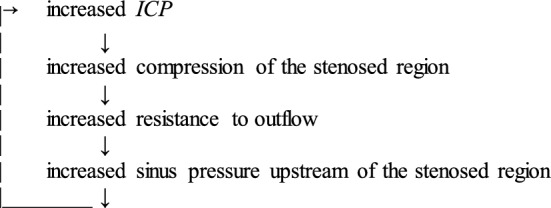


A possible consequence of this positive feedback is bistable behaviour in which *ICP* can be stable at either a high or a low value [234, 236]. Thus when *ICP* is high, the additional restriction of outflow may ensure maintenance of high *ICP* while when *ICP* is low, the reduction in the restriction can allow efficient outflow to keep *ICP* low. Such bistable behaviour provides a very convenient explanation for why a single withdrawal of CSF by lumbar puncture, which decreases *ICP,* can lead to sustained reduction and relief of symptoms for far longer than the time required to replace the amount of CSF that was withdrawn [235, 237]. A longer term reduction in *ICP* can be produced by stenting the stenosed region even if stenosis was not the primary cause.

In most interpretations of the influence of venous pressures on *ICP,* it is assumed that these pressures are opposing outflow via arachnoid villi and that the villi are the major pathway for CSF outflow. These assumptions may be correct but should be reconsidered for three reasons. Firstly it is likely that other CSF outflow pathways are also important (see Sect. 3.4 and Fig. 6), secondly a proportion of the villi may direct CSF to meningeal lymphatics rather than into the venous sinuses [112] and finally changes in pressure within the venous sinuses will be accompanied by changes in pressure in the surrounding tissue and this may well affect other routes of outflow.^29^

For adults with rigid skulls, if all important routes of CSF outflow lead from freely communicating subarachnoid spaces, increasing resistance to outflow or increasing outlet pressure should increase *ICP but* should not cause swelling of the CSF-containing spaces or shrinkage of the parenchyma [180, 238–241] (see Fig. 4 in [242]) (but see also [243] who report increased total CSF volume).^30^ The reason why the parenchyma might not shrink and the CSF spaces expand, is that the raised *ICP* is applied to the parenchyma via CSF which can itself penetrate into the parenchyma. The raised *ICP* not only increases the force driving fluid in but also increases the force on the parenchyma driving fluid out, hence the parenchymal volume remains unchanged.^31^

The condition, iIH, described above is an extreme case in which there is free communication between the ventricles and all subarachnoid spaces and all routes of outflow lead from the subarachnoid spaces. As a consequence, in strict iIH, volumes are maintained despite the increased pressure. However, factors resulting in raised *ICP* changes often lead to conditions in which there are changes in the distribution and volume of CSF (see footnote 30). These include ventriculomegaly as described in the following section.

## Ventriculomegaly

Ventriculomegaly is expansion of the cerebral ventricles. This can be dramatic, leaving the cortex as a thin shell of tissue pressed against the skull (see [244–246] and many examples in [247]). In infants ventriculomegaly can occur without gross loss of parenchymal tissue as the skull can expand (see Sect. 9.1) allowing for ventricular enlargement without reduction in cortical volume. However, while the parenchymal volume may continue to increase as the infant grows, the cortex becomes deformed, spreading out into a thinner layer. This can have consequences for long structures like axons and blood vessels that cannot easily be stretched. Ventriculomegaly and hydrocephalus in infants are considered in Sect. 9.1.

In adults with a rigid skull, the volume of the cranial contents, i.e. the sum of the volumes of the blood together with the vessel walls, CSF, ISF, intracellular fluid and solids of the brain tissue, must be the same as the volume of the space available within the skull, an observation whose importance was recognized by both Monro and Kellie [248]. Since both liquids and solids in brain tissue are almost incompressible (but see footnote 25), addition of an excess of either would increase the pressure pushing the cerebellar tonsils into the foramen magnum. This obstructs veins and CSF pathways causing further increase in pressure and further obstruction with fatal outcome. Thus it is evident that ventriculomegaly must be associated with a reduction in volume of something else within the cranium (see [249] for possible caveats). Something else could be:

- to a small extent, blood within the cerebral vasculature;
- the parenchymal framework, i.e. cellular loss, shrinkage of cells (loss of cell water) or loss of cellular components, e.g. myelin;
- CSF in subarachnoid spaces;
- ISF in the parenchyma.

Because blood volume within the vasculature in the adult human cranium is only about 60 mL (4% of the total volume see Fig. 2), decreases in this volume alone cannot compensate for the much larger volume changes of other components that occur in ventriculomegaly (or in oedema or hemorrhage). Indeed it has been proposed that, rather than being of benefit, compression of blood vessels can lead to decreased blood flow sufficient to produce ischaemia [250, 251].

Loss of brain cells or cell components can lead to ventriculomegaly and increase in volume of the subarachnoid spaces. For many years, the excess CSF seen in Alzheimer's disease or Parkinson's disease has been ascribed to the filling of space freed by brain atrophy. There is little or no suggestion that the remaining parenchyma is compressed or pressed against the skull or that *ICP* is raised. Thus, in these disorders, the cell loss is considered primary with the accumulation of CSF a consequence, occurring only to fill the available space and maintain the *ICP* needed for CSF outflow. The ventriculomegaly seen in SHR spontaneously hypertensive rats has also recently been shown to be associated with loss of brain tissue without any apparent changes in the production rate of CSF or in resistance to CSF outflow [252].

Whether cell damage and cell loss associated with ventriculomegaly are causes or consequences has been controversial for many years (see e.g. [242, 253–255]). Cell loss is also now part of the theory (see Sect. 5.2.2) that ventriculomegaly in hydrocephalus results from damage by increased pulsatility (i.e. increased variations in pressure in the lateral and IIIrd ventricles) during the cardiac cycle. There is general agreement that cell damage and loss, especially in white matter, does occur in the late stages of hydrocephalus [253, 256–260]. The resultant decrease in cell volume then allows for a substantial portion of the ventriculomegaly. The challenge of deciding whether ventriculomegaly results from accumulation of CSF forcing enlargement or from ventricular enlargement leading to CSF accumulation remains one of the most difficult aspects of clinical management of patients presenting with dementia and ventricular enlargement.

Some reduction in volume of the subarachnoid spaces is likely to accompany ventriculomegaly except when this results from brain atrophy. This is considered in Sect. 9.2.1.1. Likewise it has been suggested that reduction in the volume of the parenchyma by expulsion of ISF may occur in ventriculomegaly. This is considered in Sect. 9.2.1.2.

### Continued outflow of CSF from the ventricles in ventriculomegaly

Ventriculomegaly requires that, at least temporarily, the rate at which CSF is being added to the ventricles must exceed the rate at which it is being lost. The effect of such a difference in rates can be seen dramatically in the first few hours following experimental block of the cerebral aqueduct [23, 261, 262] and hence CSF outflow. However, as has been noted repeatedly (see e.g. [263]), in the longer term, the difference between the rates of addition and loss of CSF must be very small, otherwise the ventriculomegaly would soon exceed the entire volume of the skull. For example, Bering & Sato noted in 1963 [264] that, in infants whose ventricles were enlarging at 10 to 15 mL day^−1^ as a result of block of normal outflow, the CSF production rate was still more than 300 mL day^−1^, i.e. almost all of the CSF produced was escaping by some other route.

## Hydrocephalus

Strictly defined, hydrocephalus is any disorder in which [265–267]

- there is a defect in the handling of CSF and.
- this mishandling causes ventriculomegaly.

This commonly accepted definition excludes accumulation of CSF primarily in the subarachnoid spaces even when that is caused by altered handling of the CSF. Others prefer to use a more inclusive definition in which the mishandling causes excess accumulation of CSF in the ventricles or the subarachnoid spaces or both. Hydrocephalus with CSF accumulation primarily in the subarachnoid spaces seen when the skull can expand as in fetuses and infants is called external hydrocephalus (see e.g. [86, 268–271]). Hydrocephalus with accumulation in the ventricles is then called internal hydrocephalus though often the "internal" is implied rather than stated.

Defects that directly affect handling of CSF include occlusions of the cerebral aqueduct or the outlets from the IVth ventricle, obstructions in the basal cisterns, occlusions of the cribriform plate, and increases in pressures in the venous sinuses or the meninges, all of which affect CSF outflow. They also include changes in CSF secretion by the choroid plexuses. There are, in turn many potential causes of these defects including choroid plexus papilloma, congenital malformation of pathways, tumours, infections including meningitis, subarachnoid hemorrhage, and trauma. This review does not seek to evaluate the origins of the primary causes of the changes in CSF handling but rather to describe the changes in the physiological processes involved. Readers interested in different perspectives are recommended to see [272–275].

Hydrocephalus is such a broad concept embracing various different aspects that there is little hope of finding a single adequate description for all its forms (see e.g. [246]). However, the different aspects can be used for classification into subforms. Perhaps the most important division is between hydrocephalus in infants, whose skulls are expanding, and in adults, whose skulls are rigid. Within each of these types, hydrocephalus has been divided into non-communicating and communicating. This distinction dates back to the early description by Dandy and Blackfan [197, 238, 276]. They found in non-communicating hydrocephalus that if phenolsulphonephthalein was injected into the cisterna magna it appeared rapidly in urine and in the lumbar sac, but when injected into a lateral ventricle it appeared at these locations only very slowly. By contrast in communicating hydrocephalus they found that phenolsulphonephthalein appeared rapidly when injected into either the cisterna magna or the ventricles. These observations have been confirmed by others (see e.g. [277]). No one would now choose to use phenolsulphonephthalein as a marker in quantitative measurements of CSF flow because it is a substrate for specific transporters in the brain [278], but that does not alter the force of the qualitative distinction.

Non-communicating hydrocephalus arises when CSF flow through or out of the ventricles is obstructed. The most obvious form of this type of hydrocephalus is seen when the cerebral aqueduct is blocked. Dandy and Blackfan [238] achieved this by inserting a cotton plug into the IVth ventricular end of the aqueduct. They achieved long-term, chronic block in dogs but were not able to achieve block with long term survival in cats or monkeys.^32^

Communicating hydrocephalus is still too broad a concept to describe as a disorder with a single cause. Rather than making a strict division between communication and non-communication it is more useful to consider the variations in sites of obstruction and in extent of communication. At one extreme there are conditions with free communication through the ventricles and the cranial and vertebral subarachnoid spaces but with defects in outflow, examples of these being pediatric external hydrocephalus (see Sect. 9.1) and adult iIH (see Sect. 7). At the other extreme there are conditions with no communication via the normal routes between the lateral and third ventricles and the subarachnoid spaces but with no defects in outflow routes as with complete aqueductal stenosis. In between, there are conditions with varying degrees of restriction of flow within the brain, potentially at different locations blocking access to some spaces and not others.

The word 'hydrocephalus' has only rarely been used to describe the excess of CSF accompanying brain atrophy as in Alzheimer's disease or Parkinson's disease (see Sect. 8). Even then the accumulation of excess fluid has been called 'hydrocephalus ex vacuo' to differentiate it from hydrocephalus proper [279, 280].

**Fig. 9:**
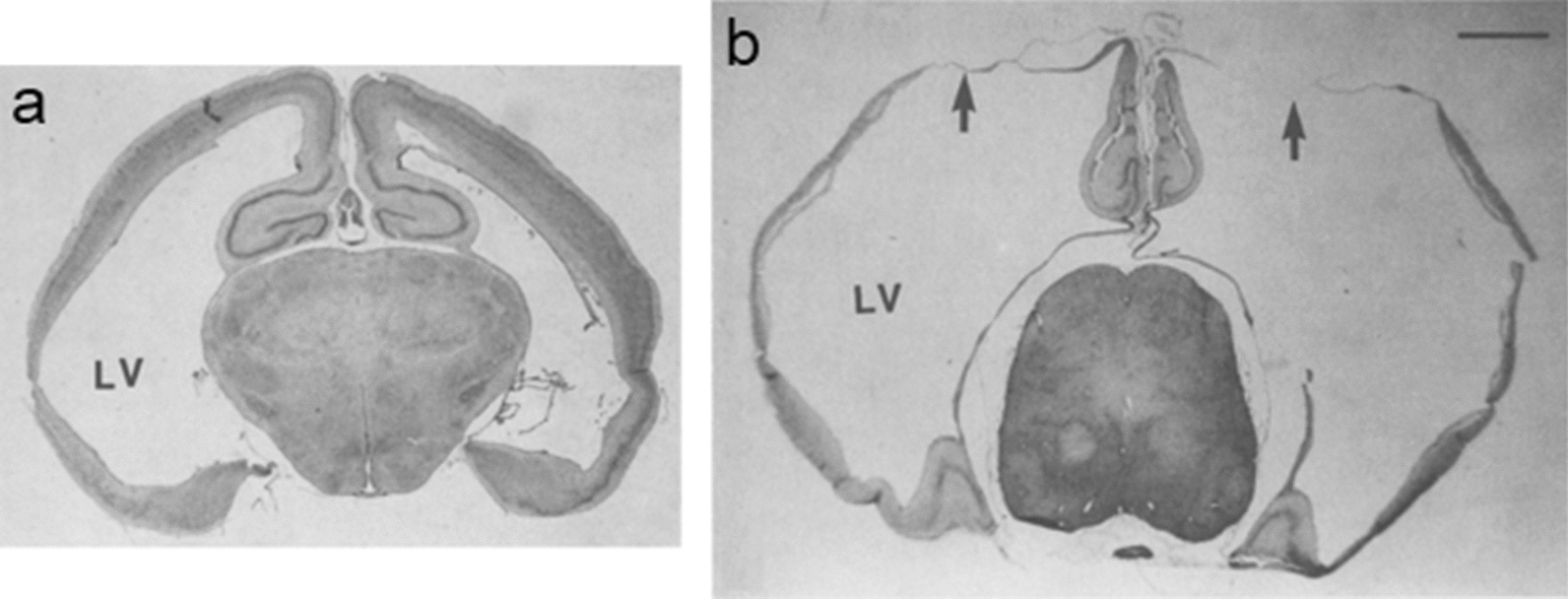
Coronal sections at the level of the parietal cortex of H-Tx rat brains **a** 10 and **b** 30 days after birth. These rats have aqueductal stenosis and other defects. Scale bar = 2 mm. The arrows in **b** indicate the thinnest portions of the cortex where the thickness is ca. 10µm. Reproduced from [245] with permission

### Pediatric and fetal hydrocephalus

Many, perhaps most cases of pediatric^33^ hydrocephalus are discovered during exploration, often using ultrasound imaging, to determine the reason for a larger than normal head. The enlargement may be a consequence of raised *ICP* resulting from an excess of CSF. An early account based largely on autopsy findings was given by Russell [281] and Weller and colleagues have described examples of the pathology (pediatric and adult) [274]. There is now a modern compendium on pediatric hydrocephalus, largely illustrated with MRI images [247]. It can result from a great variety of causes including intraventricular hemorrhage, congenital aqueduct stenosis, myelomeningocele and brain tumors [247, 272, 275].

**Fig. 10:**
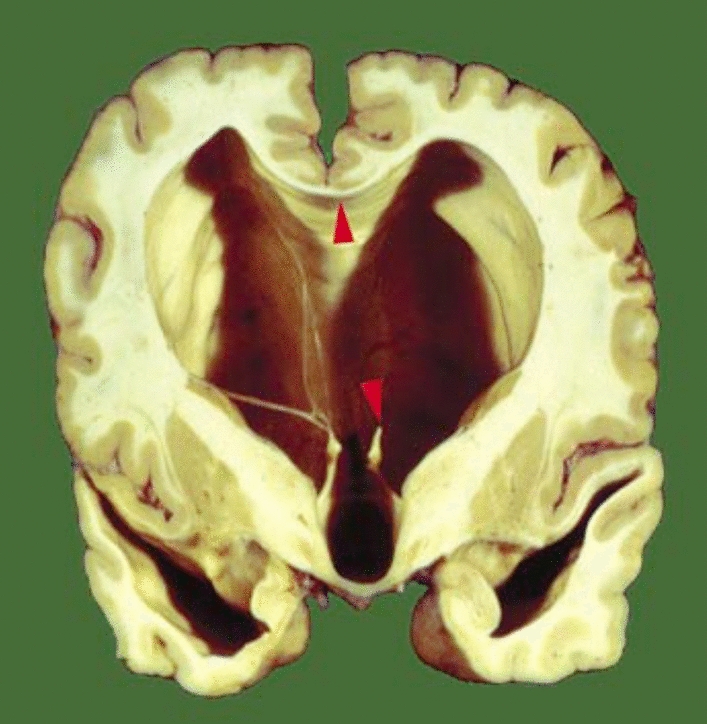
Image of a coronal slice of the brain from a young adult with chronic, untreated infantile-onset hydrocephalus. Red arrowheads indicate the greatly narrowed corpus callosum (top) and fornix (bottom). These normally contain many nerve fibres, hence the narrowing indicates substantial white matter loss. Image reproduced with permission from [259]

If left untreated, the extended ventricles in internal hydrocephalus can occupy most of the space inside the skull (see Figs. 9 and 10). Indeed in early work on pediatric hydrocephalus it was sometimes detected by the ability of light from a bright source to pass all the way through the head ([244], (see Fig. 7 in [282]). Despite there being excess CSF, in the absence of rare pathologies of the choroid plexuses, CSF secretion rate is generally believed to be in the normal range or even somewhat reduced [283–286].

There are many examples in humans and animals of genetic changes associated with abnormalities in early brain development and with ventriculomegaly (see [272, 287–291]. However, it is at present still uncertain whether (see discussion in [272]):

- the ventriculomegaly results directly from changes in the development of the parenchyma reducing its volume, in which case CSF accumulates to fill the void, which is normally not called hydrocephalus;
- the genetic changes may be linked to the handling of CSF which in turn induces the ventriculomegaly which would be hydrocephalus; or
- combination of both (see [292]).

The H-Tx rat is an animal model with inherited congenital ventriculomegaly with onset in utero and gross ventricular swelling in neonates (see Fig. 9). It has been used to study hydrocephalus because the swelling results at least in part from aqueduct stenosis that produces obstruction of CSF flow [176, 245]. Flow studies showed that there is ~ 2.3 times larger resistance to outflow of fluid infused into the ventricles than that infused into the cisterna magna [176], consistent with aqueduct obstruction. However, closure of the cerebral aqueduct in the H-Tx rat is also associated with reduced glycoprotein secretion from the subcommissural organ and abnormal cortical development (see [293–295]. Hence the ventriculomegaly is not solely due to CSF obstruction but also to complex genetic factors affecting cortical development. Consistent with this complexity is the effect of CSF shunt treatment in H-Tx neonates, which shows only partial restoration of cortical normality [296].

As now expected, the early stages of the ventricular swelling in H-Tx rats, up to neonatal 10 days, occurred with no significant difference in total pressure between the ventricles and the cisterna magna (see Sect. 5.3). Furthermore, there was little elevation of *ICP,* consistent with much of the ventriculomegaly corresponding to normal increase in head volume and decrease in subarachnoid space volume. By 30 days there was collapse of subarachnoid spaces, increase in *ICP* (no measurement was possible in the collapsed cisterna magna) and substantial head enlargement. Despite the aqueductal stenosis there is still substantial outflow. The route for this is unknown, but an obvious speculation is that it is across the thin and possibly oedematous parenchyma.

#### External hydrocephalus

External hydrocephalus occurs where there is free communication between and throughout the ventricles and subarachnoid spaces. It is produced either by increased resistance of outflow pathways from subarachnoid spaces to blood and/or lymph or by increased pressure at the outlet for one of these pathways. Because the skull expands to accommodate the excess CSF, there may be little elevation of ICP and, provided something eventually provides an adequate route for CSF outflow and limits or arrests the changes in volume, there may also be little consequence for the brain [246, 268, 270, 297, 298]. Indeed, this is sometimes called benign enlargement of the subarachnoid space [299], benign extra-axial fluid collections [246] or benign external hydrocephalus. In the corresponding condition in adults, iIH (see Sect. 7), there are no large volume changes in marked contrast to those changes seen with infants, highlighting the importance of the restraining influence of the rigid skull [210, 215, 216, 300, 301]. The importance of this can also be demonstrated in experimental systems, see Sect. 9.1.4.

So called ‘arrested external hydrocephalus’ may be more common than internal hydrocephalus in infants though this is rarely specifically diagnosed^34^, treated or studied [270, 297, 299, 302]. The arrest requires that during growth there is either a change in an existing outflow route (altered resistance or outlet pressure) or development of a new route. The nature of the changes has not been established (but see Sect. 9.2.2.1).

#### Pediatric non-communicating hydrocephalus

When there is obstructed outflow of CSF from the ventricles, CSF will accumulate there and become depleted downstream of the obstruction leading to parenchymal deformation and ventriculomegaly. Whenever this is substantial, the ventricles will have a larger surface area and the cortex will have a smaller thickness than normal. However, in pediatric hydrocephalus, cortical volume may remain nearly normal because the skull expands so increasing the total space available.

The data presently available suggest that in pediatric hydrocephalus the ventricular total pressure (= ventricular fluid pressure) may be at least several mmHg higher than normal for infants ("normal" being however substantially less for infants than for adults [303]). It should also be noted that normal infant activities, e.g. sucking and crying, markedly increase pressure and are sufficiently common to increase the average *ICP* over time [304, 305](see also [306]). Thus, single time point measurements, which in most studies are likely to have been obtained when the infant was quiet, may well have underestimated the average pressure. Presumably the increase in *ICP* above normal leads to the more rapid expansion of the skull that is a principal characteristic of pediatric hydrocephalus.

There do not appear to have been any measurements made of fluid pressure in the cortical subarachnoid spaces. In regions where the parenchymal framework presses against the outer meninges, pressure in the interstitial fluid, *ISFP,* may be substantially less than *ICP* while the stress on the parenchymal framework may be correspondingly greater (see Sect. 5.5). As discussed further in connection with adult non-communicating hydrocephalus (see Sect. 9.2.1), the reduced fluid pressure in the subarachnoid space and reduced *ISFP* are likely to be important in providing an alternative route for CSF to leave the ventricles.

#### Pediatric communicating hydrocephalus

There may be many different causes of pediatric communicating hydrocephalus (see e.g. [210] for references). Experiments to test the two proposed mechanisms considered here might provide important information about the underlying pathology.

In the first proposed mechanism, pediatric communicating hydrocephalus results from obstruction to CSF flow between the cisterna magna and sites of outflow from the cortical subarachnoid spaces [307]. This can be called communicating hydrocephalus because the CSF can traverse the cisterna magna and vertebral subarachnoid spaces to reach the lumbar sac. However, in order for CSF from the ventricles to reach a cortical site of outflow it must not only traverse the cisterna magna but also flow through the basal cisterns to reach either an outflow route along a cranial, e g. olfactory, nerve or the enlargements of the cortical subarachnoid spaces adjacent to major cerebral arteries. Any obstruction of flow along these pathways would be expected to have different effects on total stress and fluid pressure in the parenchyma and cortical subarachnoid spaces. An increase in the pressure in the ventricles, *ICP*, will be transmitted throughout the brain via fluid flow and deformation of the parenchymal framework and thus will rapidly increase total pressure/stress everywhere inside the skull. By contrast, fluid pressure upstream of the obstruction, including in the ventricles, will be increased while that downstream will be decreased.^35^ The stress on the framework will be greater downstream of the obstruction as the parenchyma is pulled up against the skull and meninges. The increased total pressure throughout the brain relative to pressure outside the head would result in progressive enlargement of the head whilst the reduced downstream fluid pressure relative to ventricular pressure would provide an increased fluid pressure gradient from ventricles to sites of outflow. The possible parenchymal deformation accommodating this combination of pressure changes includes ventriculomegaly (compare Sect. 9.2.1). Although this proposal is plausible, there is little experimental evidence to support or refute it because a) fluid pressures have not been measured and b) at present the existence of an obstruction in the basal cisterns can be confirmed only by invasive means, e.g. use of an endoscope as in [308]).

In the second proposed mechanism, increases in venous pressure in infants increase *ICP* by a small but sufficient amount to lead to expansion of the CSF spaces and the skull [215]. In infants, as discussed in Sect. 8.1.1, external hydrocephalus is often associated with elevated venous pressure. However, if there is any outflow route leading from the parenchyma to e.g. meningeal lymphatics, outflow by that route would allow fluid pressure to be lower in parenchyma than *ICP* leading to parenchymal compression and ventriculomegaly, i.e. to a form of communicating hydrocephalus (compare Hakim's proposal discussed in Sect. 9.2.2.3.1).

#### Development of experimental hydrocephalus with and without a rigid skull

The importance of a rigid skull in the development of hydrocephalus has been investigated using adult cats with hydrocephalus induced by injection of kaolin into the cisterna magna [309, 310]. Results showed that:

- If the skull and dura were intact, modest ventriculomegaly developed with an apparent plateau within 3 weeks.^36^
- If a substantial part of the dorsal skull was removed and the dura incised immediately before the induction of the hydrocephalus, there was much greater enlargement of the ventricles (and of the head) with no evidence of a plateau.

The plausible interpretation of these findings is that the constraint imposed by the skull and dura raised *ICP* sufficiently to develop an outflow route that allowed outflow to balance CSF production. Without the constraint, CSF was able to accumulate progressively without increasing *ICP* sufficiently to increase CSF output to match its rate of production.

#### Insights into the underlying pathology of pediatric hydrocephalus from current methods for its management

There can be no doubt that surgical interventions have improved the prospects for infants with hydrocephalus. There was much greater mortality before the development of practical shunts that allow CSF to be drained out of the brain [311, 312]. Insertion of a shunt, commonly ventriculoperitoneal, has allowed substantial recovery of ventricular size, rethickening of the cortical layer, an end to the excessive head expansion and recovery of neurological function. However, recovery of cortical volume and shape has often been incomplete with normal tissue replaced by gliosis or scar tissue [250, 253, 313].

Preservation of cortical volume in the early stages of pediatric hydrocephalus is consistent with there being no loss of neurons and the possibility of preserved neural connections. Permanent neurological consequences can be minor if the cortical deformation can be arrested or corrected sufficiently early [244, 253, 259, 272, 280, 311, 312, 314–318]. As Lorber put it in 1968 [244]:

" Of 28 consecutive cases treated between 1963 and 1966, 24 survive, and 16 are developing normally. These include children whose heads freely transilluminated in infancy prior to operation or whose head had to be punctured to allow delivery.

" The proportion of children with superior intelligence in children treated for extreme hydrocephalus in infancy is not less than that in the general population, as long as their hydrocephalus was not associated with myelomeningocele and their operation was not delayed beyond six months of age.

" Even the most extreme degree of hydrocephalus in young infants is no contraindication to early operative treatment."

More recent assessments confirm that highly favourable outcomes from surgical interventions are possible though perhaps less frequent than suggested by Lorber [272, 312].

The choice of treatment is often between ventriculoperitoneal shunting, which allows CSF to drain out of a lateral ventricle to the peritoneum, and ETV in which a hole is opened in the floor of the III^rd^ ventricle allowing CSF to flow between the III^rd^ ventricle and the interpeduncular cistern [222, 319–321]. Shunting has the advantage that it provides a pathway for removal of CSF from the head and thus has a high success rate for reducing cranial CSF volume. It has the disadvantage that the shunts may repeatedly need adjusting or replacement, with a lifelong commitment to potentially expensive and certainly inconvenient care. ETV has the major disadvantage that it only allows CSF to shift from the ventricles to the interpeduncular cistern which may or may not be in communication with sites of outflow via other basal cisterns with an adequate route for CSF outflow.^37^ (For further consideration of these issues, see [222, 319–321] and references therein).

In adult as in pediatric hydrocephalus the increased amount of CSF in the ventricles is the result either of an increase in rate of production of CSF or of a decrease in its outflow from the ventricles. Certainly the production rate has been shown to be increased substantially in choroid plexus hyperplasia [286], choroid plexus papilloma [158, 284, 322] or following hemorrhage [323, 324]. In addition there are isolated cases where increased CSF production from unknown causes has been documented [325–327]. However, in almost all cases where hydrocephalus persists for more than a few days, it is the processes governing CSF distribution, CSF outflow or both that appear to be altered. Regardless of whether or not CSF production rate changes, after an initial transient period, CSF outflow itself must become closely matched to CSF production (see Sect. 4).

### Adult hydrocephalus

Most hydrocephalus cases in young adults result from obvious precipitating insults such as hemorrhage, tumours, infection or traumatic damage causing obstruction to CSF flow. Such cases are called secondary hydrocephalus. Depending on the site of obstruction, secondary hydrocephalus can be either non-communicating or communicating. It is frequently accompanied by raised *ICP*, sometimes to levels requiring urgent intervention.

In cases of adult-onset hydrocephalus where there are no obvious precipitating insults, little elevation of *ICP* is usually seen. In such cases, the condition is called normal pressure hydrocephalus, NPH. It is in these cases that the distinction between hydrocephalus and atrophy leaving space for CSF accumulation can be most difficult.

**Fig. 11:**
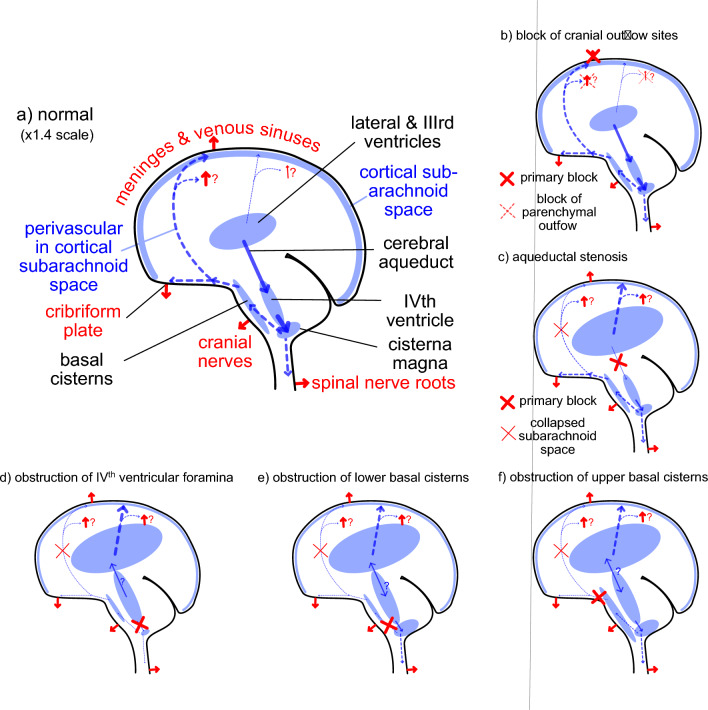
Schematic indications of CSF flow routes and outflows expected if normal flow is blocked at various sites. **a** Normal flows with no obstructions. **b** iIH where outflow from cranial subarachnoid spaces is hindered. There is increased *ICP* but no changes in ventricular or subarachnoid volumes, see Sect. 3. **c** Aqueductal stenosis leading to: ventriculomegaly (lateral and III^rd^ ventricles) see 9.2.1". **d** Obstruction at foramina of Luschke and Magendie leading out of the IVth ventricle. Ventriculomegaly now also involves the IV^th^ ventricle (see Sect. 8.2.1). c) and d) are examples of 8.2.1. **e** and **f** are examples of communicating hydrocephalus where CSF can flow from the lateral ventricles to the vertebral subarachnoid spaces. The proportion of CSF flows from IV^th^ ventricle to III^rd^ ventricle or to the cisterna magna is unknown (see Sect. 8.2.2). In **e** and **f**, the basal cisterns between the obstruction site and the cisterna magna are expected to be swollen. In **e** where the block is closer to the cisterna magna endoscopic third ventriculostomy (ETV) connecting the IIIrd ventricle to basal cisterns would be expected to reduce ventriculomegaly while in **f** where block is further from the cisterna magna and beyond the interpeduncular cistern (see text) ETV would only connect spaces already in free communication and would be expeced to have little effect (see Sect. 8.1.5.1). Important common expected features in all of c)-f) are periventricular oedema and a reduction in the flow of CSF by normal routes to the cortical subarachnoid spaces which then have reduced volume and fluid pressure, see Sect. 9.2.1.1. providing a basis for a gradient of fluid pressure to drive flow through the parenchyma (see Sect. 9.2.1)

A schematic indication of the consequences of obstructed flow in various locations is given in Fig. 11.

#### Non-communicating hydrocephalus

Many instances of adult hydrocephalus are associated with obvious obstructions to CSF flow between the lateral ventricles and the cisterna magna, e.g. at the cerebral aqueduct (see Fig. 11b) or the outlets from the IVth ventricle (see Fig. 11c). Such obstructions will produce rapid and extensive ventriculomegaly unless and until there is a new efflux route for CSF circumventing the block. If the block is at the level of the cerebral aqueduct there are only three possible new routes for CSF outflow.

- The first route involves pressure-driven flow across the choroid plexuses. This would be manifest as a decrease in rate of CSF production. However, the pressure required in experimental animals to reduce this rate to a noticeable extent has been shown to exceed any seen in the ventricles, i.e. CSF production rate is found to be independent of *ICP* [33].
- The second route allows flow through an opening that develops through the wall of a ventricle (lateral or third) closely adjacent to the lining of a subarachnoid space allowing fluid to escape. There is experimental evidence for such a route in rats [53, 328, 329] but also evidence against such a route in rhesus monkeys [277]. There is also an MRI study which suggests such openings in humans with hydrocephalus but not in normal subjects [330]. Further studies are required.^38^
- The third possible route for outflow from the ventricles is via the parenchyma. It is generally accepted that solutes and fluid can enter the parenchyma as seen during the development of periventricular oedema ([331–337] and for further references [338]). This oedema may extend up to 2-3 mm from the ventricular surfaces but does not include the entire parenchyma.^39^

However, there are two difficulties that substantially delayed acceptance of the idea that CSF can flow out of the ventricles via the parenchyma. Firstly the interstitial spaces of the parenchyma normally provide a high resistance to flow (for general discussion see [339] and for aspects relevant to the CNS [37, 340, 341] with further discussion in [4, 92]). Secondly something must keep the interstitial fluid pressure (see section 5.2.2) below the ventricular pressure so that there is a driving force for the flow out of the ventricles.

Resistance to flow in the first 0.5–3 mm from the ventricular surface will be greatly reduced in hydrocephalus due to periventricular oedema (compare [37, 173, 342]). Further within the parenchyma, flow will continue along perivascular spaces that provide much lower resistance than do the interstitial spaces in the rest of the parenchyma [4, 92, 302, 343]. Such perivascular spaces extend to the cortical surfaces from within a few millimeters of the ventricular surface.^40^ The combination of periventricular oedema and perivascular spaes may well provide the low resistance route required for outflow of CSF from the ventricles via the parenchyma to either the cortical subarachnoid space or possibly to lymph vessels in either the meninges or at the base of the skull^41^ (see Sect. 3.4.4).

If CSF is to flow from ventricles to parenchyma and thence out of the brain, there needs to be a fluid pressure gradient. Thus, *ISFP* in the parenchyma must be lower than fluid pressure in the ventricles but greater than fluid pressure at the sites for outflow from the brain. It should be noted that the fluid pressure in the cortical subarachnoid spaces is not known; the measurements that have been made [175, 177–179] have been of total pressure (see Sect. 5.2.2) which may differ from the fluid pressure if the framework of the parenchyma has contacts with the outer meninges.

Block of the usual outflow route from the ventricles presumably initially increases the total pressure within them to above the total stress, fluid pressure plus solid tissue stress, in the parenchyma and cortical subarachnoid spaces which plausibly will drive the changes in volumes including ventriculomegaly. As discussed, the excess in pressures required to drive enlargement is likely to be small. When the volumes are no longer changing rapidly, the *total* pressure (stress) will be the same throughout (see Sect. 5.3) but there will still be a net flow driven by the gradient of *fluid* pressure. The observation that the total pressure throughout the ventricles and patent subarachnoid spaces and total stress in the parenchyma (together with by inference the total stress in collapsed subarachnoid spaces) are all the same once the parenchyma has shifted and/or deformed in no way precludes the existence of a fluid pressure gradient that can move CSF around or out of the brain. There is no need for a total pressure gradient to maintain ventriculomegaly.

As discussed in Sect. 4, because of the constraints of the skull, extensive ventriculomegaly in adults requires a reduction in volume of either the subarachnoid space or the parenchyma. These scenarios are discussed in the next two sections.

##### Reduced volume of CSF in subarachnoid spaces

In non-communicating hydrocephalus, because the normal outflow routes from the ventricles are occluded, ventricular pressure is increased and that pressure is transmitted as the total stress to the parenchyma and to subarachnoid spaces (see 5.3"). Initially, CSF outflow from the subarachnoid spaces is maintained (or even increased) via normal routes. However, CSF inflow to those spaces is reduced and so the volume of CSF and fluid pressure within the spaces will decrease until contact of the parenchyma with the outer meninges sufficiently increases solid tissue and total stress to oppose further decrease in volume.

Apparently there has never been an attempt to assess quantitatively the reduction in size of the subarachnoid spaces. As described by Rekate et al. [222] such assessment would be difficult.

**Fig. 12:**
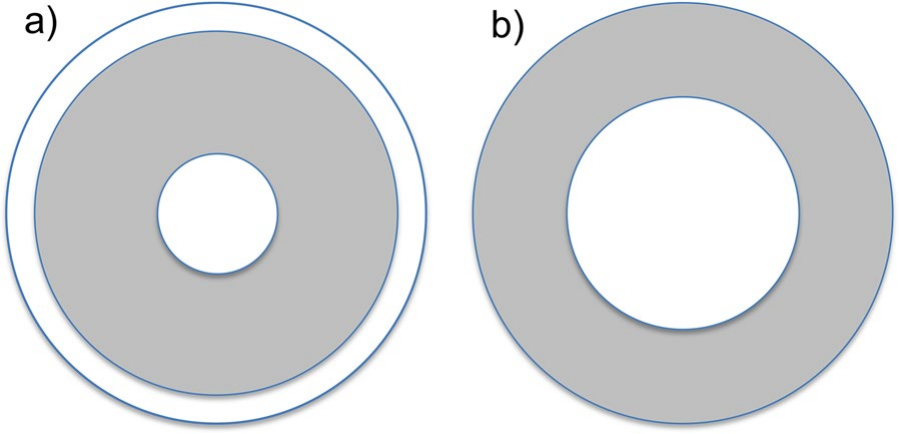
Diagrams to illustrate how substantial ventriculomegaly could occur without compression of the parenchyma. Cross sections through the middle of spheres representing CSF (shown in white) and parenchyma (shown in grey). **a** In the normal situation, the volume of CSF in the ventricles is 35 mL, the volume of the parenchymal tissue is 1160 mL and the volume of CSF in the subarachnoid spaces is 215 mL (see Fig. 2 and footnote 2). **b** The situation if all the CSF is redistributed and becomes contained inside swollen ventricles; CSF volume is still 250 mL and the volume of the parenchymal tissue remains at 1160 mL. The parenchyma is not compressed though its thickness is obviously decreased (compare discussion of the similar diagram in [222])

Normally the volume of CSF in the ventricles of an adult, 20- 33 mL, is much less than that in the cranial subarachnoid spaces including the basal cisterns, ~ 215 mL (see Fig. 2 and footnote 2). Thus a reduction in CSF volume in the subarachnoid spaces could allow substantial ventriculomegaly. Indeed it has been suggested that the rapid changes in ventricular volume that can be seen with shunt failure and repair are unlikely to be possible by any other mechanism [222]. An illustration of the principle is shown in Fig. 12. The major easily visible changes if such compensation occurs would be ventriculomegaly accompanied by a flattening of the cerebral foldings against the dura and skull with collapse of the sulci just as seen in acute obstructive hydrocephalus in rhesus monkeys [262].

**Fig. 13:**
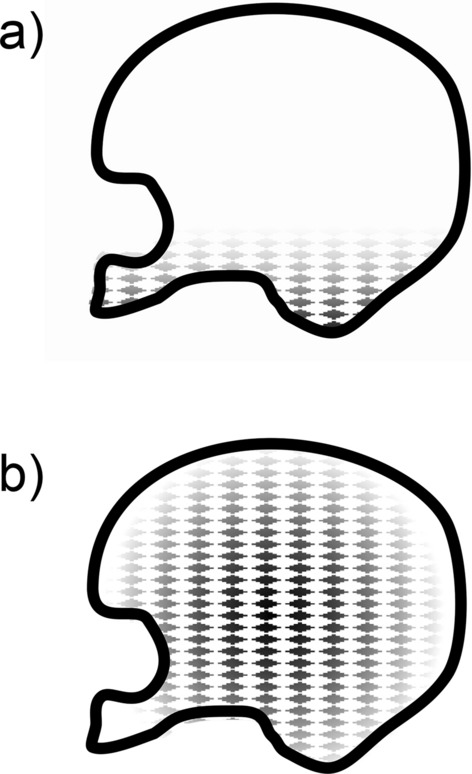
Schematic drawings depicting left lateral view cisternograms of the head of a 4 year old child with congenital aqueductal stenosis following injection of I^131^-radiolabed human serum albumin into the lumbar region. The dashes indicate sites of location of the radiotracer, the darker ones indicating higher concentration. The cisternogram in panel a) was obtained when a non-functioning shunt was in place and there was extensive ventriculomegaly. The cisternogram in panel b) was obtained after replacement with a working shunt. As shown in **a** the I^131^-radiolabed human serum albumin has been able to reach as far as the basal cisterns but there appears to be little radiotracer above this level suggesting collapse of the subarachnoid space. As indicated in **b** after successful replacement of the shunt and reversal of at least part of the ventriculomegaly, injected I^131^-radiolabed human serum albumin can now be detected well above the basal cisterns and so is able to reach the subarachnoid spaces over the entire cortex. Diagrams based on cisternograms in [344]

These observations make it likely that much of the early stages of the development of acute ventriculomegaly following obstruction of the cerebral aqueduct or outlets from the IVth ventricle is at the expense of the volume of the subarachnoid spaces. Evidence that the subarachnoid spaces are reduced in size and the parenchyma presses against the meninges has also been obtained in cases of advanced hydrocephalus where there is human congenital aqueductal stenosis. Furthermore shrinkage of the ventricles after shunting allows the subarachnoid spaces to reopen (see e.g. [344]) (see Fig. 13). The extreme upper limit for ventriculomegaly at the sole expense of the subarachnoid spaces would be an increase in volume from 33 mL to about 240 mL as indicated in Fig. 12.

Compression of the subarachnoid spaces in acute non-communicating hydrocephalus has been generally accepted for many years and, possibly as a consequence, there has been little work using more modern techniques to assess its extent. Similar compression in normal pressure hydrocephalus, is, however, still under active consideration as will be discussed in Sect. 9.2.2.2.

##### Reduced volume of the parenchyma.

The alternative to reduction in the volume of the subarachnoid spaces to allow ventriculomegaly is reduction in the volume of the parenchyma. There is general agreement that in advanced stages of hydrocephalus there is extensive loss of cellular material in white matter (see e.g. Figures 5, 10) and that this loss is largely irreversible [250, 256–258, 281, 334, 336, 345, 346]. With shunting there can be some restoration of cortical mantle thickness, and thus plausibly of cortical volume, but this is not entirely recovery of normal tissue [257, 347–349].

There is less agreement about whether there is reduction in parenchymal volume in the early stages of non-communicating hydrocephalus and if so whether it reflects 1) exclusion of ISF from the tissue (with no compensating increase in intracellular water) or 2) loss of entire cells or cellular constituents, e.g. lipids and proteins in white matter [254]. Assuming parenchymal volume does decrease in the early stages, the major arguments in favour of the view that this decrease is due to exclusion of ISF are: a) that any alternatives to loss of ISF would be too slow to account for the time course of changes after blockage or insertion of a shunt; b) that the reduction in thickness of the cortical mantle is reversible following shunting; c) that cellular loss would lead to much more functional loss than seen in the early stages; and d) that early shunting is able to restore functional loss that does occur [222]. Obviously an ultimate upper-limit of parenchymal volume reduction by ISF exclusion would be the volume of ISF initially present, normally about 20% of the total, i.e. ~ 230 mL (see Fig. 2).

While the hypothesis that ISF is expelled from the parenchyma in the early stages of non-communicating hydrocephalus is attractive, there have been remarkably few studies of how cortical parenchymal volume and water content change during the development of ventriculomegaly. Most of these have failed to detect any decreases (see e.g. [333, 350–353]). There have, however, been two notable exceptions.

In the first case, Penn and Bacus [255] inspecting CT scans observed that the x-ray density of the tissue increased with distance from the ventricular surface and interpreted this as a lower water content which might plausibly account for a reduction in parenchymal volume. However, as other factors can change the x-ray density, this result needs to be confirmed using techniques that directly determine water (as noted by Marmarou [354]). Higashi et al. [352] were unable to detect any change in water content, but apparently no other studies have been forthcoming largely because the measurements are very difficult for very small tissue samples.

In the second case, Del Bigio and Bruni [355] studying silicone-induced hydrocephalus in rabbits found that the specific gravity of small samples was increased, except at the ventricular surfaces. This increase implies water loss. Similarly using MRI and kaolin induced hydrocephalus in rats Massicotte et al. [356] found decreased apparent diffusion constant (ADC) for water, initially throughout the brain but subsequently, in periventricular regions, replaced by an increase in ADC. They interpreted the results as indicating brain compression in grey matter but compression followed by development of oedema in white matter. However, in subsequent work Del Bigio and Enno [357] working with kaolin induced hydrocephalus in rats found that whereas the ISF volume was reduced, the total water content, intracellular and extracellular, was increased, i.e. the parenchyma was expanded not compressed. Del Bigio and coworkers [358] again found that the total water content in the parenchyma was increased.

In conclusion, there is little evidence from these experimental studies to support the idea that the parenchyma shrinks due to water loss. Indeed there is some evidence suggesting that there is water gain [357, 358]. Gain has also been inferred from calculations made of cortical volume in the H-Tx rat with congenital aqueductal stenosis (see Sect. 9.1). In these rats there was gross ventriculomegaly and cortical thinning (see Fig. 9). When serial sections were inspected, despite the thinning, the calculated cortical volume was increased [245]. Furthermore water made up a larger proportion of the cortical mass [359], Thus in HT-x rats there is evidence for accumulation rather than exclusion of water.

Ventriculomegaly stretches the ventricular surfaces in regions of convex curvature. The stretching of the framework parallel to the surface is accompanied by compression of the framework perpendicular to it (see e.g. [360–362]). Further from convex ventricular surfaces there may be compression in all directions. Description of these effects has been the object of extensive theoretical work with the development of poroelastic models evaluated by finite element methods [80, 242, 360–364]. For critical discussion and references to the extensive, more recent work see [365–368]. However, while simulations based on plausible mechanical properties for the framework and realistic geometry for the parenchyma predict the periventricular oedema [338, 361–363, 369–371], they have not predicted substantial net removal of ISF possibly because they have not accounted for the (as yet unmeasured) fluid pressures in the subarachnoid spaces. Furthermore, to date, none of the modelling has incorporated realistic descriptions of perivascular spaces. Nor has the modelling allowed for multiple routes of outflow. There is much to be done.

#### Communicating hydrocephalus

Communicating hydrocephalus is a catch-all category. The features always present are:

- ventriculomegaly with reduction in the combined volume of the cranial subarachnoid spaces and the parenchyma; and
- communication between the ventricles and *vertebral* subarachnoid spaces.

Ventriculomegaly combined with increased total volume of the subarachnoid spaces within the cranium is normally interpreted as being the result of cerebral atrophy.

In most examples of adult communicating hydrocephalus (*other than secondary hydrocephalus, see* Sect. 9.2) there is:

i. Nearly normal ventricular pressure (*ICP*);
ii. Reduced volume of the cortical subarachnoid spaces;
iii. Decreased pulsatile CSF flow in time with the cardiac cycle through the foramen magnum between cranial and vertebral subarachnoid spaces, but increased variations in *ICP* (called increased pulsatility) and increased pulsatile flow through the cerebral aqueduct;
iv. Reflux of solutes through the aqueduct into the third and lateral ventricles.

In more detail:

i. Any example displaying the first of these is called **normal pressure hydrocephalus (NPH)**.

ii. The prime candidate for a defect causing communicating hydrocephalus with shrinkage of the cortical subarachnoid space is a blockage in the pathway for CSF flow from the cisterna magna to cranial sites of outflow (see Fig. 3 and Sect. 9.2). Blockade at these sites would still allow transfer of markers and tracers between ventricles and lumbar sac. If outflow routes from the cranium were instead blocked, then the dorsal subarachnoid spaces would be expected to expand. Thus, as has been noted frequently, the results are not consistent with blockage of arachnoid villi being the critical factor inducing hydrocephalus [180, 238–242, 372, 373].^42^

Indeed, lack of increase in resistance to outflow from the cortical subarachnoid spaces may be important for allowing continued CSF inflow from the ventricles to these spaces and thus to sites of outflow from the brain. The normal route out of the ventricles, which is blocked, may be replaced by fluid flow through the parenchyma as in non-communicating hydrocephalus (see Sect. 9.2.1). This is because the relatively low fluid pressure in the cortical subarachnoid spaces creates a fluid pressure gradient driving fluid out of the parenchyma into the spaces. Removing fluid from the parenchyma reduces *ISFP* which in turn creates a fluid pressure gradient from ventricles to parenchyma.

Sometimes the volume of the basal cisterns is reduced but, in a substantial proportion of instances of NPH, they are expanded (see Sect. 9.2.4). In such cases they are then at least partially isolated from some of the normal pathways to sites of CSF outflow.

iii. In communicating hydrocephalus it is commonly observed that the pulsatile flow of CSF through the foramen magnum is decreased while the pulsatile flow through the cerebral aqueduct is increased. Changes in *ICP* are also increased. As described in Sect. 3.3.1 the pulsatile flows and pressure changes are driven by the cardiac and respiratory cycles. Whenever the cranial subarachnoid spaces are compressed it is harder for pressure changes to drive CSF into and out of these spaces. As a consequence, a larger pulse pressure develops and the aqueductal stroke volume increases [177–179]. The retrograde movement from IVth towards IIIrd ventricle during diastole becomes large enough to transfer solutes in the reverse direction.

iv. Net flow of fluid through the aqueduct is the difference between the much larger flows into and out of the fourth ventricle during a cycle period. Normally the net flow is from the IIIrd to the IVth ventricle, but in instances of NPH this net flow may be reversed. High MW radiopharmaceuticals injected into the lumbar sac reach and are retained in ventricles of patients with communicating hydrocephalus but not of normal controls [89] ([90]. Similarly when markers were injected into the cisterna magna of dogs made hydrocephalic by producing a silastic plug in the basal cisterns (sparing the cisterna magna) [146],^43^ there was free access of the tracers into the ventricles and the vertebral subarachnoid spaces but much less rapidly the cortical subarachnoid spaces [146] [90, 374, 375]. In these studies when the markers had high MW there was accumulation of the markers in the ventricles, not just access, as if there were retrograde CSF movement carrying the markers from the cisterna magna to the lateral and IIIrd ventricles with the CSF then penetrating the parenchyma leaving the markers behind. Whether the CSF production in the IVth ventricle, which is prevented from reaching cortical sites of outflow, can account for both outflow via spinal nerve roots and retrograde flow through the aqueduct is still unknown.

MRI studies in humans using gadobutrol have found that in NPH compared to normal controls there is substantially increased reflux of intrathecally added markers into the ventricles [91] which reflects at least increased pulsatile flow and may indicate reversed net flow. At least in principle, the increased pulsatile flow could account not only for access of markers but also, to some extent, persistence of marker in the ventricles when the concentrations in the cisterna magna are decreasing (see Appendix C for discussion of the persistence of marker concentrations in dorsal subarachnoid spaces). MRI-PC studies (see Footnote 11 in Sect. 3.3) suggest that there is reversal of the net flow [77, 78, 81, 376] but the error margins are such that the evidence is not conclusive [82]. Thus the best evidence for retrograde flow is still that obtained in the 1970s. Further studies are needed.

##### The two-hit hypothesis

Many individuals who develop adult NPH without an obvious precipitating cause have large heads. Based on this observation, Bradley [86, 377] suggested that the adult hydrocephalus is a result of two successive defects. The first hit is external pediatric hydrocephalus which never required treatment and often was not even recognized as anything other than a larger than usual head. During infancy, CSF outflow was brought into balance with CSF production by development of some other route for outflow. The two-hit hypothesis proposes that this route is via the parenchyma along perivascular spaces (now often called glymphatics) allowing outflow either to subarachnoid spaces or meningeal lymphatics. The second hit in "old-age" is relative ischaemia taking place in white matter. This leads to loss of myelin impairing the parenchymal outflow route thus resulting in frank (internal) hydrocephalus. Bradley suggests that the loss of relatively hydrophobic myelin affects aqueous pathways through white matter that become lined with a more hydrophilic surface, so immobilizing water adjacent to the surface and impeding fluid movement.

There are two difficulties with this two-hit proposal. Firstly, it does not explain why ventricular pressure is not then elevated, i.e. it does not explain why the hydrocephalus is "normal pressure". Secondly in hydrodynamics, surfaces are usually said to bring to zero the velocity of fluid flowing immediately adjacent to the surfaces (the so-called no-slip condition [378]) regardless of whether the surfaces are hydrophilic or hydrophobic. Furthermore, myelin loss should increase the volume fraction of water in white matter (at least in regions of periventricular oedema) and this would be expected to decrease rather than increase resistance to outflow. A possible counterargument to these objections that would preserve the important features of the two-hit hypothesis would be to suggest that the changes in white matter somehow interfered with flow in the perivascular spaces.

##### Reduction in subarachnoid space volume

Ventriculomegaly in adults always requires that some other intracranial volume is decreased. In many instances of communicating hydrocephalus there is reduction in volume of the subarachnoid spaces above the Sylvian fissure. As indicated in Fig. 11 and Sect. 9.2.2.3.1, this is expected if there is obstruction to CSF flow from ventricles to these spaces. However, little is known about the total change in subarachnoid space volume including that of the basal cisterns. Obviously this information is important if the mechanisms involved are to be properly understood. For arguments that reductions in total subarachnoid space volume occur and are important see [222]. The tacit assumption made in all theoretical studies of communicating hydrocephalus is that the decrease in total subarachnoid space volume within the cranium is not sufficient to allow the observed ventriculomegaly in the early stages. However, this assumption was made before it was appreciated that the CSF volume in the cranium is much larger than stated in textbooks (see Fig. 2 and associated footnotes). Further experiments to determine the actual changes are needed.

##### Reduction in volume of the parenchyma

If in adult hydrocephalus, as commonly assumed but rarely if ever confirmed experimentally, the increases in ventricular volume in adult communicating hydrocephalus exceed the decrease in cranial subarachnoid space volume (and blood volume), the volume of the parenchyma must somehow be decreased. There is general agreement that in cases where the communicating hydrocephalus is long standing there is extensive loss of cellular materials (compare Sect. 9.2.1.2), i.e. much of the reduction occurs by brain atrophy induced either by the hydrocephalus or by the factors causing the hydrocephalus. The following considers the earlier changes where it is hoped that the volume changes and the loss of function might be reversible.

Attempts to explain the early events associated with parenchymal contraction have focused on parenchymal volume reduction being due to expulsion of ISF [242, 361, 363, 368, 369] or to damage to the parenchyma resulting from decreased cerebral compliance and increased amplitude of ventricular pressure pulses driven by the cardiac cycle [379]. The first of these alternatives is considered in Sect. 9.2.2.3.1, the second in Sect. 9.2.3.

###### Expulsion of ISF from the parenchyma

Obviously if parenchymal contraction is solely by exclusion of ISF, the volume of ISF initially present, normally about 20% of the total parenchymal volume or ~ 230 mL, sets an ultimate upper-limit to the volume reduction. In what follows in the rest of this section, it will be assumed that much or most of the ventricular expansion is at the expense of the parenchyma and, in the early stages of hydrocephalus, the reduction in parenchymal volume results from expulsion of ISF.

Theoretical descriptions of parenchymal contraction in communicating hydrocephalus have become increasingly more complicated. The old idea that communicating hydrocephalus results simply and directly from block of arachnoid villi as the principal outflow route of CSF is not tenable. Even if the arachnoid villi provided the sole route for outflow, that mechanism would produce iIH as discussed in Sect. 3 [180, 238–241].

In a landmark paper, Hakim et al. [242] proposed a mechanism that would produce parenchymal volume reduction (see Fig. 4 in [242]. They noted that block of CSF outflow from the subarachnoid spaces and the accompanying increase in *ICP* would increase parenchymal pressure (as in iIH, see Sect. 3). Furthermore, if there were to be a route allowing ISF to leave the parenchyma without entering the ventricles or subarachnoid spaces, then parenchymal pressure could then exceed the outlet pressure for the route from the parenchyma and ISF would be expelled. However, their proposal required there to be sustained elevation of ventricular and subarachnoid pressures. Furthermore, in the form they proposed, it failed to account for the demonstrable movements of fluid between the parenchyma and the CSF containing spaces.

The simplest scheme (closely related to the schemes considered by Levine [369] and by Peña et al. [361]) that is qualitatively consistent with NPH is obstruction of CSF flow between the cisterna magna and the sites of CSF outflow from the cortical subarachnoid spaces. Those routes of outflow are replaced by penetration of CSF into the parenchyma, producing periventricular oedema which greatly reduces the resistance to flow through the first 2 to 3 mm of the parenchyma. From there the CSF/ISF enters perivascular spaces and flows to cortical subarachnoid spaces or to lymphatics in either the meninges (perhaps in the parasagittal region) or at the base of the skull. Proposals for routes of outflow to lymphatics include those outlined in the glymphatic [92] and iPAD [103, 380] hypotheses (see [3, 4] for further discussion). Apparently, there has not been any comparison of meningeal outflow of CSF in the presence and absence of hydrocephalus.

As discussed in the context of non-communicating hydrocephalus in Sect. 9.2.1.1, the block of the normal route for CSF flow to the cortical subarachnoid space but continued outflow from it reduces both volume and fluid pressure within the space. This may lead to extraction of fluid from the adjacent parenchyma which shifts and/or deforms into the space freed by CSF outflow, so allowing ventricular enlargement without requirement for an increase in ICP.

#### Pulsatility and parenchymal damage leading to loss of tissue

The alternative to CSF accumulation driving ventricular enlargement is ventricular enlargement creating space that becomes filled with CSF, so maintaining or restoring *ICP* (see Sect. 5.3).

This concept has been considered by Bering [381] and subsequently Greitz (see [180, 382]) who proposed that ventriculomegaly can result from increased variations in pressure in the ventricles, so-called pulsatility. These variations are known to be produced by changes in blood volume in the brain during the cardiac and respiratory cycles (see Sect. 3.3.1). The increased pulsatility in communicating hydrocephalus is thought to result from a decrease in compliance defined as the change in volume divided by the change in pressure when fluid is added.^44^

Increased pulsatility is observed in communicating hydrocephalus with the amplitude of pulse pressure even exceeding *ICP*. However, it has not been established that the increased pulsatility causes the hydrocephalus; rather it may be a consequence of it. Firstly, whenever there is a reduction in subarachnoid space volume as a consequence of hydrocephalus, a decrease in compliance and increase in pulsatility will occur regardless of the cause of that hydrocephalus. Thus, a correlation between communicating hydrocephalus and increased pulsatility provides no evidence for pulsatility as cause rather than effect. Secondly, it has not been explained how changes in pressure or the small pressure gradients could damage the parenchyma. Such damage would be expected to result from sheer stresses (imagine tearing or ripping) or gradients of pressure (variations in space rather than in time) but not from changes in compression as the materials of the parenchyma are nearly incompressible. The pulse pressure occurs almost simultanously and with same amplitude throughout the brain with very small changes in the tissue predicted from a viscoelastic model of the parenchyma [383].

There are now good reasons for not taking this hypothesis further. Bering's main evidence, derived from studies of hydrocephalus induced by injecting kaolin into the cisterna magna of dogs, was that prior removal of the choroid plexus from one ventricle prevented swelling of that ventricle but not of the other.^45^ Linninger et al. [384] have shown by quantitative modelling that this result is expected without invoking any special effects of pulsatility on the ventricles. Further difficulties with the hypothesis as promoted by Greitz have been presented by Levine [385]:

- Many conditions such as aortic insufficiency or hypercapnia that increase pulsatility do not cause ventriculomegaly.
- cervical spinal stenosis, cerebral edema, or pseudotumor cerebri, all of which reduce craniospinal compliance do not cause ventriculomegaly;
- it provides no explanation for the rapid reduction in ventricular volume sometimes seen after shunting;
- provides no explanation for the gradients of pressure (or perhaps sheering stress) that would be needed for the pulsatility to damage the ventricular walls. It is to some extent contradicted by the observation that the pressure gradients that do occur are very small.

Perhaps the greatest attraction of the pulsatility hypothesis was that it seemed to provide a plausible alternative to the presumed idea that large ventricular pressures would be needed to produce hydrocephalus. However, the total pressure differences between ventricles and parenchyma or subarachnoid spaces required during development of ventriculomegaly are now known to be very small (see Sects. 5.3 and 9.2.2.3.1).

It should be noted that, while present evidence and argument do not support increased pulsatility as the cause of NPH, neither the evidence nor the arguments exclude parenchymal damage as the reason for parenchymal shrinkage (compare e.g. [386]).

#### Idiopathic normal pressure hydrocephalus (iNPH)

There are instances of adult hydrocephalus which display nearly normal *ICP* and a triad of functional symptoms: gait disturbance, dementia and urinary incontinence. In 1965 Adams et al. [387] reported three selected clinical cases in which these signs and symptoms were evident along with collapse of the subarachnoid space above the cortex. In these cases the ventriculomegaly was accompanied by a relatively rapid onset of dementia^46^ (compared to that in Alzheimer's disease), gait disturbance (see [388] for detailed analysis of gait defect) and, in two of the cases, urinary incontinence. (These three symptoms are now sometimes called Hakim's triad). In the selected cases, improvements in symptoms following ventriculoatrial shunts (2 cases) or a ventriculocisternal shunt (1 case) were dramatic with recovery of the patients to their normal mental states before the onset of the disorder. The incontinence was also alleviated and gait was markedly improved. Hakim and coworkers proposed that this combination of signs and symptoms is a separate type of adult-onset hydrocephalus, now called idiopathic normal pressure hydrocephalus, iNPH. They also asserted that it is of the greatest importance to be able to diagnose this form of hydrocephalus as the symptoms can be largely reversed by treatment. It remains the only form of dementia that is treatable. By inference, unreported cases in which the improvements were not so dramatic were presumed either to reflect other types of hydrocephalus or to be cases in which the hydrocephalus had persisted for too long, thus producing irreversible changes. The need remains for diagnostic criteria that can be used for deciding which patients would benefit from a shunt (or ETV) (see e.g. [386, 389]). Indeed, finding such criteria has been the stated or implied motivation behind much of the published research on adult hydrocephalus for more than thirty years, see e.g. [390, 391].

The prevalence of iNPH in the general population appears to be > 5% relative to all with dementia. It increases with age (and with the publication year of the study reporting the data) and is reported to be > 5% with reference to the total population over 80 [392–395]. Improvement in gait following shunting has been reported in as high as 70% of patients diagnosed with iNPH (see [396] for a review) though improvement of dementia has been much less impressive, at least partly because patients often have other forms of dementia simultaneously with iNPH.

There are differences between countries in the diagnostic criteria and in the nomenclature for defining a shunt-responsive type of hydrocephalus. In the United States (and to some extent Europe) [397–399] if there is ventriculomegaly, normal or slightly raised *ICP*, and at least two of the signs in the Hakim triad, positive response to insertion of a shunt is expected (see also [400, 401]). In Japan [402] emphasis has been placed on DESH (disproportionately enlarged subarachnoid-space hydrocephalus) [372, 402, 403], in which the cortical subarachnoid spaces are shrunken but the Sylvian fissure and basal cisterns are expanded. This pattern has been found to predict a positive shunt result. Both sets of criteria allow for alternative tests to predict success of intervention.^47^

## Overview and summary

Sections 2–5 present an updated and extended version of our understanding of normal aspects of production, circulation and outflow of brain extracellular fluids. Important updates include:

- Total cerebrospinal fluid (CSF) volume determined by MRI, ~ 335 mL, is substantially larger than the "textbook" value of ~ 140 mL. The earlier value was based on a single series of experiments in 1915 (Fig. 2 and footnotes 1 and 2).
- The rate of active secretion of CSF by the choroid plexuses is still an unknown proportion of the total rate of production of brain extracellular fluid (CSF plus ISF). However, the choroid plexuses probably contribute more than half (Sect. 3.1). The rest would be by active secretion across the blood-brain barrier (Sect. 3.2).
- The available data on the osmolalities of ISF and CSF compared with those of blood have been collected together, apparently for the first time, and are presented in Appendix B.
- Water moves much more freely between blood and the brain than solutes like NaCl. Unidirectional radiotracer fluxes of tritiated water are very large across the blood-brain barrier and imposed osmotic gradients lead to large net fluxes. By contrast, in the absence of maintained, imposed osmotic gradients, net water fluxes are much smaller. The steady-state distribution of water is determined by the distribution of solutes and the osmolalities of all the fluids are similar. While there are, almost certainly, movements of water directly coupled to solute transfers, these movements do not create sustained gradients of osmolality between blood and tissue fluids (Sect. 3.2.1 and Appendix B).
- There is net CSF flow from the lateral and IIIrd ventricles through the cerebral aqueduct to the IVth ventricle and then into the cisterna magna where the flow divides, some going to the vertebral subarachnoid spaces and some to basal cisterns from which a relatively small flow continues to the cortical subarachnoid spaces (Sect. 3.3). Net flow into the vertebral subarachnoid spaces is likely to be substantial, e.g. > 20% of CSF production, but will vary with posture.
- The cardiac cycle influences the movement of fluid in the brain. In systole, more blood enters the brain than leaves and CSF shifts from the brain to the vertebral subarachnoid space. These flows are reversed during diastole. The respiratory cycle has similar effects. The net flow of CSF through the cerebral aqueduct is the difference between the much (e.g. tenfold) greater flows in the forward and reverse directions. The net flow through the foramen magnum is an even smaller fraction of the forward and backward flows (Sect. 3.3.1).
- There are multiple routes for outflow of fluid from the CNS including: the extracellular spaces of cranial nerves, notably the olfactory nerve leading via the cribriform plate to lymphatics (and blood microvessels) in the nasal mucosa; spinal nerve roots; arachnoid villi allowing passage across the meninges to lymphatics and venous sinuses; and perivascular spaces connecting in some manner to meningeal lymphatics. The nasal route is dominant in mice and rats and important in sheep and Rhesus monkeys. In humans multiple routes may be important (Sect. 3.4).

Sections 5–5.5 provide an outline of the volumes, stresses and pressures normally present within the cranium. CSF in large spaces, e.g. ventricles and basal cisterns, is a liquid in which the pressure on the fluid is the same as the total pressure. The parenchyma is a composite of a framework of cells that can be considered as an easily deformed solid and ISF that fills the spaces between the elements of the framework. Intracranial pressure, *ICP*, is defined as the pressure on CSF in the lateral ventricles. In the absence of pathological restraints on movements of CSF and parenchyma, *ICP* equals the total pressure everywhere in the CNS once the effects of gravity are taken into account. When the volumes are not changing, *ICP* takes on the value which balances the strongly pressure-sensitive fluid outflow and the relatively pressure-insensitive rate of CSF and ISF production (Sect. 5.3). The extracellular fluid volume is the volume that fills the available spaces to the extent that increases *ICP* to the level required for the balance of outflow and production.

The parenchyma is a composite structure with ISF filling the spaces within a cellular framework. Thus, it is necessary to distinguish between interstitial fluid pressure *ISFP* and the solid tissue stress on the framework with total stress a weighted sum of the two (see Sect. 5.2.2). (The total stress is often called total pressure, see below.) ISF movements occur down gradients of *ISFP*. If the framework is freely suspended in and surrounded by CSF, the stress on the framework is equal to ISFP and both are the same as the pressure on the CSF which is the total pressure. However, if the framework is pressed against external solids the stress on the framework can substantially exceed *ISFP* (Sect. 5.2.2).

Gradients of total stress within the brain under normal conditions are too small to measure except in brief transients and in certain pathological conditions. By contrast gradients of fluid pressure must always exist to drive the continuing flow of CSF. "Normal" *ICP*, the same throughout the brain, does not imply that there are no gradients of fluid pressure (Sect. 5.5).

Table 1 provides an overview of what is and what is not known about the processes governing the movements of fluids (other than blood) and about changes in the shape and volume of the brain under normal conditions.

**Table 1:** Summary of the processes governing normal extracellular fluid movements (other than of blood) in the brain and changes in brain shape and volume

	Level of knowledge			
Topic	Known with some certainty	Likely	Unknown	See sections:
Fluid secretion via the choroid plexuses	Large part of net fluid entry into the brain. Active transcellular and passive paracellular components (a "leaky" epithelium)l	More than half of the total fluid entry Net rate reduced a little by increases in ventricular pressure	Actual proportion of fluid entry into the brain	3.1
Net fluid movement across the blood–brain barrier		There is likely to be some secretion into the brain	Actual proportion of fluid entry into the brain	3.2
Circulation of CSF	Small net flow superimposed on larger flows driven by cardiac and respiratory cycles Pressure differences needed to drive the net flows are < 1 mmhg		Distribution of flows in the subarachnoid spaces, e.g. Proportion of flow passing over the dorsal surface of the cortex	3.3
Fluid entry into the parenchyma	Some fluid entry via periarterial routes	Some secretion across the blood–brain barrier	Sizes of inward flows via either route?	3.3.2
Fluid exit from the parenchyma	There is normally a small net flow from white matter into the ventricles	Fluid exit via perivascular routes: some to the subarachnoid spaces, some to meningeal lymphatics	Sizes of the fluid movements via any of the routes	3.3.2
Fluid outflow from the brain	Multiple routes of csf outflow from the subarachnoid spaces including cranial nerves (prominently the olfactory, trigeminal and optic nerves), spinal nerve roots, arachnoid villi	Outflow of ISF from the parenchyma via a route that avoids mixing with CSF in the subarachnoid spaces	Actual proportions of outflow via each route (cannot infer these for humans from data for rodents) Nature of the connections between perivascular routes and lymphatics	3.4–3.4.4
Deformations of the parenchyma	Acutely almost incompressible, but shape and position within the cranium can change Given time, volume changes can occur reflecting changes in composition – e.g. gain or loss of ISF, intracellular fluid, myelin, or cells	Estimates of time courses of gains and losses of isf and of shifts between intracellular and extracellular fluids	Only very rough estimates are available for the elastic properties of the framework and how these are affected by changes in intracellular fluids	5.1–5.2.2

Sections 6–9 consider the alterations in function occurring in the production, circulation and outflow of brain extracellular fluids in the pathological conditions, intracranial hypertension, ventriculomegaly and hydrocephalus.

Intracranial hypertension (Sect. 7) occurs when outflow of fluids from the brain is obstructed or the pressures at the destinations of the outflow routes are increased. Any condition that produces intracranial hypertension without changes in CSF volumes in the adult is called idiopathic intracranial hypertension (iIH). iIH is usually accompanied by increased pressure in the venous sinuses and venous stenting can relieve the raised *ICP*, but it can still be unclear whether the raised sinus pressure is cause or effect.

Ventriculomegaly (Sect. 8) is enlargement of the cerebral ventricles for whatever reason. In adults with a rigid skull, this requires that some other volume, in practice the volume of the subarachnoid spaces or that of the parenchyma, must decrease and it also requires that transiently inflow of fluid into the ventricles must exceed the outflow. Either the accumulation of CSF in the ventricles or the physical increase in ventricular size can be cause and either can be effect. If the ventricles expand as a result of cerebral atrophy then CSF accumulates to fill the increased space and restore ventricular pressure to that required to drive out CSF at the rate at which it is being produced. Only rarely has this been called a type of hydrocephalus and even then as "hydrocephalus ex vacuo".

If, instead, CSF accumulates because outflow from the ventricles is hindered, then the (perhaps transiently) elevated ventricular pressure drives the ventriculomegaly and the accumulation of CSF is called hydrocephalus. In the longer term, regardless of whether atrophy or accumulation comes first, the difference between the rates of CSF addition and loss must be very small; otherwise the volume of the swollen ventricles would soon exceed the entire volume of the skull (Sect. 8.1). Thus, whenever the normal routes of outflow from the ventricles are blocked for more than a few hours, there must be some other route by which the CSF secreted by the choroid plexuses can escape.

Hydrocephalus (Sect. 9) in adults with rigid skulls is conventionally defined as any disorder in which mishandling of CSF causes ventriculomegaly. In infants with skulls that are growing, hydrocephalus can be more conveniently defined as any disorder in which mishandling of CSF brings about a larger than usual increase in total CSF volume. Indeed it is sometimes extended further to include any congenital disorder in which CSF volume is increased. Hydrocephalus in infants can be external, where the subarachnoid spaces are expanded, or internal, where the expansion occurs as ventriculomegaly.

Defects that directly affect handling of CSF include occlusions of the cerebral aqueduct or of the outlets from the IVth ventricle, obstructions in the basal cisterns, occlusions of the cribriform plate, and increases in pressures in venous sinuses or meninges, all of which affect CSF outflow. They also include changes in CSF secretion by the choroid plexuses.

In pediatric and fetal hydrocephalus (Sect. 9.1), those defects that increase *ICP* and produce iIH in adults bring about a pronounced increase in volume of the skull and CSF-containing spaces. These increases are driven by the increase in pressure difference between inside and outside the head. Often the subarachnoid spaces are swollen (Sect. 9.1.1). Defects that result in obstructions to CSF circulation can produce ventriculomegaly as they do in adults. Regardless, CSF accumulation can occur without a decrease in rate of growth of the parenchyma because the whole head is increasing in size. That raises the possibility that functional impairments can be avoided if adequate routes for CSF outflow can be provided.

It is traditional to subdivide hydrocephalus (internal hydrocephalus in infants) into non-communicating and communicating (Sects. 9.1.2, 9.1.3, 9.2, 9.2.1 and 9.2.2). In the former, solutes are unable to access the lumbar sac from lateral ventricles whereas in the latter, they can. In non-communicating hydrocephalus, the obstruction to CSF flow is in the cerebral aqueduct or in the various ventricular foramina. In communicating hydrocephalus, there may still be an obstruction to flow but this probably occurs somewhere between the cisterna magna and cranial sites of outflow (cranial nerves, arachnoid villi or routes leading to meningeal lymphatics). Obstructions at these locations would still allow fluid movements from ventricles to vertebral subarachnoid spaces.

In adults in either non-communicating or communicating hydrocephalus, ventriculomegaly is usually accompanied by shrinkage of at least portions of the cortical subarachnoid space. This brings the parenchyma into closer contact with the meninges. Wherever that occurs, the parenchymal framework will bear a larger portion of the forces acting across the space and the fluid pressure within the space will be reduced. That provides a pressure gradient driving fluid from the parenchyma into the subarachnoid space. This in turn reduces *ISFP* within the parenchyma thus providing a pressure gradient driving fluid into the parenchyma from the ventricles.

In non-communicating hydrocephalus, CSF must still be able to leave the ventricles at a rate close to its rate of production by the choroid plexuses – otherwise the ventricles would expand at much greater rates than those observed. With stenosis of the cerebral aqueduct, the likely route is via the parenchyma. Resistance to fluid flow through interstitial spaces is normally very high, but in parenchymal regions where in non-communicating hydrocephalus there is periventricular oedema this resistance may be greatly reduced. Onward flow through the parenchyma may be possible via perivascular spaces which extend from close to the ventricles to the brain surfaces (Sect. 3.3.2).

In both communicating and non-communicating hydrocephalus, substantial ventriculomegaly is only possible if the volume of the subarachnoid spaces or the volume of the parenchyma or both is reduced (Sects. 8, 9.2.1.1 and 9.2.1.2). Reduction in the volume of subarachnoid spaces is likely to occur because CSF flow routes into these spaces are obstructed more than outflow routes from them, but this reduction may not be sufficient to provide for the ventriculomegaly even in the early stages of hydrocephalus. Reduction in the volume of the parenchyma does occur in the later stages of hydrocephalus – and is then irreversible. The possibility that parenchymal volume can be reduced reversibly by expulsion of ISF (Sects. 9.2.2.3 and 9.2.2.3.1) has been explored in many theoretical studies, experimental evidence being sparse.

The idea that parenchymal damage resulting from increased pulsatility explains ventriculomegaly was initially put forward in 1962. Pulsatile variations in total pressure (pulsatility) do occur in the brain as a result of changes in blood volume. Furthermore these variations are substantially larger in communicating hydrocephalus. However, it does not follow that this increased pulsatile pressure produces increased damage to the periventricular parenchyma. Physical changes to the parenchyma require pressure gradients or sheer stresses, while the pulsatile changes in total pressure occur throughout the brain with any gradients too small to account for ventricular wall damage. Furthermore, there is now a plausible explanation for the development of ventriculomegaly without change in *ICP* (see Sect. 9.2.2.3.1). Similarly the CSF flow that must persist throughout hydrocephalus can be driven by relatively small gradients in fluid pressures. Unfortunately, measurements of fluid pressures that would allow quantitative testing of theoretical models are not yet available.

Idiopathic normal pressure hydrocephalus (iNPH) is the condition where there is a combination of normal pressure hydrocephalus together with Hakim's triad symptoms, these being gait disturbance, dementia and urinary incontinence (Sect. 9.2.4). As documented in the original reports, surgical treatment could produce dramatic functional improvements. However, even in the original reports, it was clear that a diagnosis of iNPH was not sufficient to predict whether or not any treatment of the disorder(s) would be successful. It remains a major challenge to establish diagnostic criteria that can prove useful in predicting if treatment of NPH will or will not be beneficial.

## Conclusions

The processes that determine CSF production, circulation, volume and outflow and intracranial pressure, *ICP*, were described in the 1960s and 1970s (see e.g. [7]).The view then was that most CSF enters the brain via pressure-insensitive secretion by the choroid plexuses, flows through the ventricles and subarachnoid spaces acquiring a small contribution from ISF, and then leaves the brain via pressure-sensitive flow through arachnoid villi to the venous sinuses. *ICP* was stated to be the pressure in the brain that matches CSF outflow to production and total CSF volume to be that which fills the available spaces sufficiently so as to achieve *ICP*. CSF flow through the brain was presumed to be driven by pressure gradients with higher pressures in the ventricles also serving to keep the ventricles inflated.

While this description is still broadly correct it needs extending in at least five important respects:

- There are several routes for CSF outflow which are species dependent and may vary with circumstances. In many species, the extracellular spaces of the olfactory nerve provide a prominent route leading ultimately to lymphatics in the nasal mucosa. In addition, an unknown but probably substantial fraction of outflow occurs from the vertebral subarachnoid spaces. The fraction that drains to the cranial venous sinuses in rodents is close to 0 but in humans is likely to be larger though still much less than 1. The fractions draining by the various routes may vary with anaesthesia or posture and almost certainly change in the development of hydrocephalus.
- CSF volume in humans, now measured using magnetic resonance imaging, is at least twice as large as the textbook value [7]. (This has implications for the source of the excess fluid in the ventricles in adult hydrocephalus.)
- The net flow of CSF from the ventricles is superimposed on a much larger (e.g. 10 to 20 fold) pulsatile CSF flow oscillating between the cranium and the vertebral subarachnoid spaces. This pulsatile flow serves the important function of allowing changes in cranial blood volume during the cardiac and respiratory cycles.
- The composite nature of the parenchyma was not considered in the classical view. The parenchyma consists of a solid, deformable framework of cells with the intervening gaps filled with ISF. This structure has implications for understanding the forces, i.e. the stresses and pressures, within and imposed on it. Conventional pressure sensors measure the total force per unit area i.e. the total stress not the fluid pressure.
- As closely as it can be measured, the total stress is normally the same throughout the brain (after allowing for gravity). This is the expected theoretical result from the physical properties of the parenchyma and the resistances to CSF flows. However, gradients of total stress can occur if the parenchyma is hindered from moving and deforming, e.g. by being pressed into the foramen magnum or less dramatically by being pulled into contact with the meninges.

These extensions to the classical view have substantial implications for the understanding of ventriculomegaly and hydrocephalus. The primary mishandling of CSF in hydrocephalus produces transient gradients of total stress but sustained gradients of fluid pressure. The total stress gradients persist only as long as the parenchyma is being moved and deformed. The fluid pressure gradients, however, must be sustained to dispose of the continually produced CSF. Only very small fluid pressure gradients are required for flow along normal routes.

Hydrocephalus was originally viewed as resulting from a block of CSF outflow from the subarachnoid spaces to venous sinuses. In the presence of continuing CSF production this block was thought to lead to excess ventricular CSF accumulation. However, it is now clear that, since adults have rigid skulls, obstruction of outflow from the subarachnoid spaces leads to raised *ICP*. When this occurs with no perceptible change in CSF volumes it is called idiopathic intracranial hypertension (iIH). iIH is accompanied by raised cerebral venous sinus pressure, but whether this is a cause or a consequence of raised *ICP* is often uncertain*.* Obstruction of CSF outflow in infants whose skulls are still growing leads to an increased rate of expansion of the skull with or without enlargement of the ventricles.

In both adults and infants, ventricular enlargement due to CSF mishandling involves interference with CSF circulation. The site of this interference determines whether the hydrocephalus is non-communicating with CSF outflow from the ventricles obstructed or communicating where CSF can move between the ventricles and vertebral subarachnoid spaces. In the latter case, obstruction occurs at sites hindering normal delivery from the cisterna magna to cranial sites of outflow.

Originally the view was that the ventriculomegaly of hydrocephalus resulted from an increase in intraventricular pressure. However, because the measured pressure increases appeared to be too small, an alternative view was put forward. This view stated that the ventriculomegaly results from damage to the parenchyma produced by increased pulsatile variations in intraventricular pressure. There are indeed increases in pulsatile pressure, but these are the same throughout the cortex with no large gradients that could plausibly cause parenchymal damage. Current understanding is that only small transient gradients of total pressure are necessary to bring about movement and deformation of the parenchyma including changes in the size of the ventricles. The most important changes may indeed be in the subarachnoid spaces, with obstructed flow of CSF into these spaces and continued CSF outflow from them leading to a reduction in subarachnoid space volume. The parenchyma may then shift into the space leading to ventriculomegaly.

There must still be gradients of fluid pressure from the ventricles to sites of outflow and sufficiently low resistance routes to allow outflow of the continuing CSF production. There will be a gradient of fluid pressure across the parenchyma because a) ventricular fluid pressure is maintained by CSF production and b) there is continuing outflow of fluid from the subarachnoid spaces but block of some of the routes by which the fluid normally reaches those spaces. A fluid pressure gradient with no total pressure gradient is possible because the framework is pulled into contact with the meninges increasing the solid tissue stress at that surface.

Normally the resistance of the interstitial spaces in the 2–3 mm layer of parenchyma adjacent to the ventricles is too high to allow outflow of the ventricular CSF producted, but in hydrocephalus this resistance will be greatly reduced by periventricular oedema. Beyond this layer, flow is possible via perivascular spaces that connect with the subarachnoid spaces.

There are variations in presentation of hydrocephalus and in its response to treatment because of the large number of different places in the brain where mishandling of CSF can occur and the lack of understanding of how changes in parenchymal volume and shape produce reversible and irreversible functional damage. Perhaps the most troubling aspect is the lack of diagnostic criteria that can predict when treatment of hydrocephalus can or cannot prevent further progression or even a reversal of functional deficits. Reasons why success has not been greater may be the still limiting resolution of current imaging techniques and the shortage of suitable measurements of fluid pressures and of subarachnoid space volumes. It is also clear that successful treatment becomes increasingly difficult as the condition progresses. Criteria for determining when functional deficits have become irreversible are needed.

The physical and physiological principles underlying the normal control of fluid volumes and pressures provide plausible explanations of the physical changes in the development of hydrocephalus. However, there are still many details to be established and so further experimental evidence is required. This includes determination of:

- The sites of obstructions (apparently those in the basal cisterns are particularly difficult to detect);
- The changes in volume of the various subarachnoid spaces;
- Fluid (as opposed to total) pressures;
- The mechanical properties of the cellular framework of the parenchyma (i.e. How easily is it deformed); and
- The changes in the routes of CSF (and possibly ISF) outflow.

However, even more importantly much more needs to be known about the relation between the structural changes and functional loss.

## Figures and Tables

**Fig. 14:**
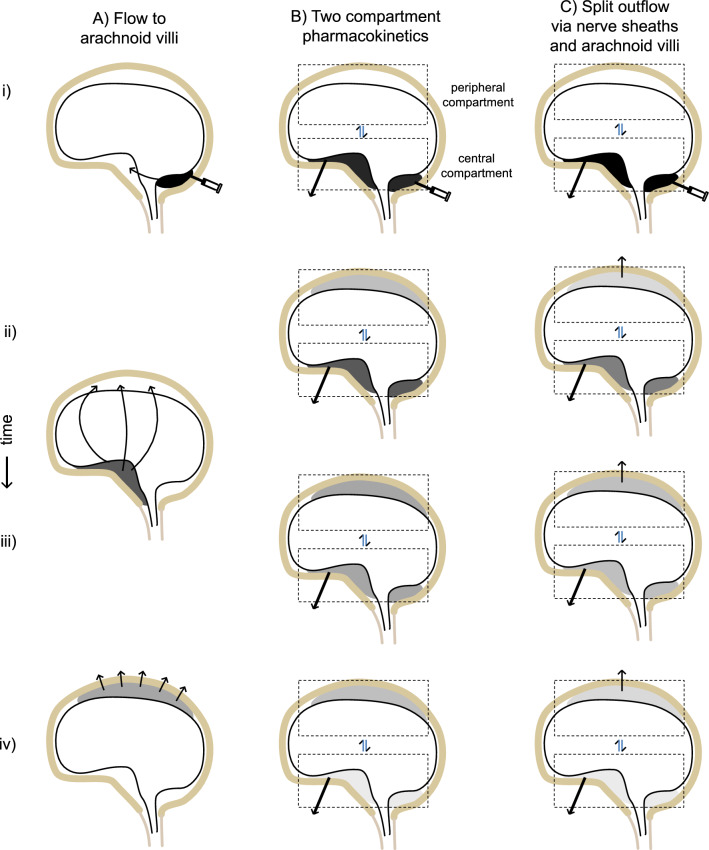
Three scenarios illustrating possible movement of markers through the CSF containing spaces of the brain after injection into the cisterna magna. **A** The traditional model in which marker is distributed by flow of CSF to the dorsal subarachnoid space and eliminated via arachnoid villi. i) Initially, it flows into the other basal cisterns. ii/iii) From there it flows via the perivascular spaces running in the subarachnoid spaces over the surface of the brain to the dorsal subarachnoid spaces. iv) The marker leaves the brain in the CSF outflow through the arachnoid villi to reach blood in the venous sinuses or meningeal lymphatics. **B** A two-compartment pharmacokinetic model in which the dorsal subarachnoid spaces constitute the peripheral compartment and the cisterna magna and sites of elimination are parts of the central compartment. Marker in the peripheral compartment must return to the central compartment for elimination from the brain. i) Initially, the injected marker rapidly distributes through the central compartment. ii) At this stage, marker concentration in the central compartment exceeds that in the peripheral compartment and so marker is transferred from central to peripheral compartment. Elimination from the brain also decreases the concentration in the central compartment. iii) At this stage, central concentration has fallen to equal the, until now increasing, peripheral concentration. iv) Thereafter, concentration in the peripheral compartment exceeds that in the central compartment. **C** An intermediate model in which there is rapid elimination from the ventral spaces and some, but slower, elimination from the dorsal spaces and slow transfer between ventral and dorsal regions. i) The injected marker rapidly distributes through the central compartment. ii) Elimination from the ventral subarachnoid spaces occurs rapidly via cranial nerves (and spinal nerve roots), marker is carried by relatively slow flow to dorsal subarachnoid spaces and slow elimination occurs from these spaces via arachnoid villi. There may or may not be return of marker from the peripheral to the central compartment iii) Ventral concentration continues to decrease and dorsal concentration increases until the rate of transfer of marker into the dorsal spaces falls below the rate of dorsal elimination out of the brain. iv) Ventral elimination continues to be faster than dorsal such that at some time point the dorsal concentration then starts to exceed that in the ventral spaces. Note that in A) all elimination of marker occurs via outflow dorsally via the arachnoid villi while in B) no elimination occurs via that route. Both scenarios can explain the sequence of concentrations observed experimentally. C) shows a compromise that allows elimination via multiple routes including the arachnoid villi

## Data Availability

No new data are reported in this review. There is no data to share.

## Appendix A Volume flux and flow, the Staverman-Kedem-Katchalsky equation and Starling's mechanism

The volume flux, *J*_*V*_, across a barrier like a capillary wall is the net volume of fluid transferred across the barrier per unit time per unit area by all the fluxes of solutes and water. This applies regardless of whether the mechanisms by which the solutes and water move are best described as being by diffusion, by flow, or, as is almost always the case, by a process which has features of both.^48^ It has become conventional to describe* J*_*V*_ in the language of irreversible thermodynamics. In these terms the volume flux is driven by the differences of hydrostatic pressure, *ΔP*, and osmotic pressure, *Δπ,* across the wall [404, 405],

$$J_V = L_P \left( {\Delta P - \Delta \pi } \right) = L_P \left( {\Delta P - RT\sum_i {\sigma_i \nu_i \Delta c_i } } \right)$$

where *L*_*p*_ is the filtration coefficient, Δ*P* the hydrostatic pressure difference, Δπ the osmotic pressure difference, *R* the gas constant, *T* the temperature, σ_i_ the reflection coefficient for the *i*th solute, υ_*i*_ the number of particles added to the solution for each solute molecule, e.g. υ_NaCl_ is 2, and Δc_i_ the concentration difference for the *i*th solute.^49^ If it is desired to name this equation after individuals it should be the Staverman-Kedem-Katchalsky equation using the names of the researchers who derived it [404, 405]. This equation assumes that the fluxes of water and the solutes are passive, which means that they are not coupled to metabolic energy by any means other than the concentrations and the hydrostatic pressures. The other principal limitation of this equation is that it applies only close to equilibrium, sometimes only so close as to be of limited use (see below). In practice this equation is most useful for qualitative or semi-quantitative explanations. For further discussion of the fundamental limitations of this equation see [404, 406].

In peripheral capillaries both water and small solutes, e.g. NaCl, are highly permeable but large molecules, the colloids primarily serum albumin, are not. Thus the reflection coefficients of the colloids are indistinguishable from 1 while those of the small solutes, the crystalloids, e.g. the salts of Na^+^ and K^+^ with Cl^−^ and HCO_3_^−^, are smaller. In addition the small solutes permeate sufficiently rapidly that their concentration differences across the capillary walls are small (except for brief transients after abrupt changes in concentrations). Thus $\sigma_i \nu_i \Delta c_i$ approaches zero for each of the crystalloids and only the colloids exert an osmotic pressure across the membrane. As Starling described in 1896 [44], in peripheral capillaries the fluid composed of water and small solutes easily moves down the combined gradient of hydrostatic and *colloid* osmotic pressures, a process now called the Starling mechanism.

Starling’s [44] great contributions to the study of volume fluxes across peripheral capillary walls were the division of solutes into crystalloids, e.g. NaCl, and colloids, e.g. serum albumin, and the demonstration that the volume flux of the *water and crystalloids* was proportional to the resultant of the hydrostatic and *colloid* osmotic pressure differences. (This would now be qualified by stating that proportionality was found when the volume flux was into the tissue, see below). The derivation of the Staverman-Kedem-Katchalsky equation came some 50 years later. It says more than proposed by Starling and applies in many more circumstances than he considered.

The derivation of the Staverman-Kedem-Katchalsky equation from the principles of irreversible thermodynamics (which in turn are based on statistical mechanics) requires that the filtration coefficient *L*_*p*_ is the same whether the volume flux is driven by hydrostatic or osmotic pressure differences. No justification has been given for the introduction of different filtration constants for fluxes driven by hydrostatic and osmotic pressures by Simard and coworkers [407–410]. If there are two parallel, independent transport processes (e.g. transcellular and paracellular), they can have different filtration coefficients but for each process consideration must be given to both hydrostatic and osmotic pressure differences. Stated a bit loosely the requirement from irreversible thermodynamics is that there is a hydrostatic pressure that can balance the osmotic pressure for each route. Much of what Simard and coworkers have tried to explain using different filtration constants should instead be interpreted in terms of parallel routes and different reflection coefficients of the various solutes.

Rectification of flow (or volume flux) is said to occur when the resistance to flow in one direction is different than for flow in the opposite direction. Rectification of flow does not exist sufficiently close to equilibrium. The Staverman-Kedem-Katchalsky equation predicts that it does not exist over the entire range of the equation's validity. If rectification is observed, a more mechanistic description is required possibly incorporating barriers in series or unstirred-layers. Rectification is observed for flows across peripheral capillaries. Herring and Paterson's update of Levick's textbook [411] provides an introduction to the more complicated description that is now thought to be required for the flows that are encountered in practice across peripheral capillary walls.

By contrast to what is seen in the periphery, at the intact blood–brain barrier because the permeability to NaCl is very low and its reflection coefficient is almost 1 (see [35, 47]), water and hence most of the volume flux moves down the gradient of hydrostatic and *total* osmotic pressures. This is not the mechanism that Starling described.

## Appendix B How similar are the osmolalities of plasma, CSF and brain tissue?

If the mechanisms of choroid plexus secretion and of any secretion that occurs across the blood–brain barrier are active transport of solutes with water following osmotically, then the osmolality of CSF and ISF must be somewhat greater than that of blood plasma to provide the driving force for the water movement. Measurements of the osmolalities of blood plasma and CSF are listed in Table 2. The average over the studies with humans reporting both values are 288 mOsmol kg^−1^ in plasma and 290 mOsmol kg^−1^ in CSF with an average excess in CSF over plasma of 1.6 mOsmol kg^−1^. This suggests that lumbar CSF is nearly isosmotic with plasma. As the lumbar sac is far removed from the ventricles and the cranial subarachnoid spaces, the composition of CSF there may not be typical of cranial CSF (see e.g. [412]). Because there is relatively free exchange of water and solutes between CSF and ISF, the osmolality of lumbar CSF is likely to reflect some sort of average value for ISF rather than the osmolality of the choroidal secretion. The implication is that ISF osmolality in the human is very close to that of plasma.

The averages for the non-human studies are 298 mOsmol kg^−1^ in plasma and 304 mOsmol kg^−1^ with an average excess in CSF over plasma of 5.6 mOsmol kg^−1^. With two exceptions the authors of the non-human studies concluded that CSF is hyperosmotic to plasma. The CSF samples in these studies were sometimes obtained from the cisterna magna and sometimes from a cannula inserted into a lateral ventricle, sites much closer (literally and figuratively) to the choroid plexuses than the lumbar sac. These values are consistent with the commonly held view that the secretion of the choroid plexuses may be slightly hyperosmotic to plasma.

There is a long history of attempts to measure directly the difference in osmolality between tissues and plasma. With peripheral tissues two major difficulties were encountered: a) autolysis of tissue in the time interval required to obtain the tissue, prepare it, and make the measurement in the osmometer^50^, and b) interference by the tissue structure with the freezing/melting transitions. The latter has led many investigators to dilute dispersed samples before measurement. There has been no gold-standard procedure for preventing autolysis, though rapid freezing immediately they are removed from the animal followed by boiling the samples seems to have worked best. Using vapour pressure osmometry rather than freezing point depression appears to be simpler and more reliable for measurements on tissue slices prepared from kidneys [413] though it is still not self-evident that this completely avoids artefacts due to damage during sample preparation.

**Table 2:** Osmolalites in plasma and CSF in mOsmol kg^−1^

Plasma or serum	CSF	Species	References
289	289	Human	[414]
x	x + 4.3	Human	[415]
283	285	Human	[416]
296	296	Human	[417]
288	288	Human	[418]
287	289	Human	[419]
283.6	289.5	Human	[420]
292	292	Human	[421]
293	296	Human	[422]
298.4	304.3	Sheep (fetal)	[423]
x	x±3	Dog	[424]
296	304	Dog	[425]
295	308	Dog (newborn)	[426]
308.5	314.8	Cat	Bito, unpublished in [427]
295 ±1	301 ± 3	Rabbit	[428]
298.5	305.2	Rabbit	[427]
298.7	293.5	Rabbit	[429]
293	309	Rat	[430]
306.4	307.2	Rat	[422]
290	291	Pig	[422]

Agreement appears to have been reached for peripheral tissues (e.g. renal cortex, liver, skeletal muscle and red blood cells), that the difference in osmolality between the tissues and plasma is less than the accuracy of the measurements, i.e. less than typically 1–2 mOsmol kg^−1^ [413, 431–437] consistent with the high permeability of peripheral capillary walls to water and osmotically important solutes.

For the brain parenchyma the experimental data have still not led to a convincing conclusion. Not surprisingly the available data are for small experimental animals rather than humans. The difficulty of autolysis in brain samples remains but can be reduced by reducing temperature [438] and with vapour pressure osmometry the sample can be introduced into the instrument still in a frozen state [439]. Comparisons of the osmolalities of samples of brain tissue with those of blood plasma are shown in Table 3. All but the two studies from Arieff et al. [428, 440] found that the brain tissue is hyperosmotic to plasma. It is notable that the studies by Arieff et al. are the only ones to use boiling to prevent autolysis.

It is instructive to compare the experimental measurements of brain tissue osmolality with a rough upper estimate of the deviation from osmotic equilibrium required to drive sufficient water flux through the blood–brain barrier. If a) the volume flow per gram of tissue is known, *Q* = *J*_*V*_**A*, where A is the area of the barrier per gram of tissue and *J*_*V*_ is the volume flux across the barrier (see Eqn A-1), b) it is assumed that the water flow is driven by the difference in osmotic pressure and for simplicity that this arises from the concentration difference of a single impermeant solute (σ = 1), and c) the water permeability of the barrier, *LpRT*, is known; then the Staverman-Kedem-Katchalsky equation can be solved for the concentration difference of the solute.

**Table 3:** Comparison of the osmolalities of plasma and brain tissue in milliosmoles per kg of water, mean ± s.e.m.

Plasma or serum	Brain tissue	Species	References
297 ± 3	308 ± 3	Cat	[438]
295 ± 14*	302 ± 13*	Rabbit	[440]
295 ± 1	299 ± 6	Rabbit	[428]
298 ± 0.7	306 ± 0.7	Rat	[439]
303 ± 1	311 ± 2.8	Rat	[441]
-	309.3 ± 3.6	Rat	[442]

*These values are given as mean ± 95% confidence intervals***

$$\Delta c = {{J_v } / {L_p }}RT = {Q / {L_p RTA}}$$

An estimate of the possible net secretion rate, Q, can be taken as 0.1 µL g^−1^ min^−1^ (see point 4 in Sect. 4.1 of [2]) while the area and water permeability can be taken as 100 cm^2^ g^−1^ and 1.2 × 10^–3^ µL min^−1^ cm^−2^ mM^−1^ ([35, 39] for rats and [38] for humans, see also footnote 16 in [2]), which together lead to Δc = 0.83 mM. This represents a 0.3% difference between the osmolalities on the two sides of the barrier, which is less than the errors in the measurements of brain osmolality.

By contrast during the development of ischaemic oedema, experimental volume flows are ~ 200 µL g^−1^ h^−1^ = 3.3 µL g^−1^ min^−1^ which would require a difference in osmolality of about 22 mOsmolal which is similar to the values that have been measured experimentally [34].

This calculation can be viewed in another way. If the normal value of Δ*c* were 8 mOsm kg^−1^ (≈ 8 mM) as suggested by many of the studies in Table 3, then the volume flow would be 1.2 µL g^−1^ min^−1^ which for a 1400 g human brain would be at least 5 times larger than the rate of production of CSF. While not inconceivable, it is difficult to see how a flow from parenchyma to CSF of this magnitude would have eluded detection. Thus this comparison suggests that further experiments are required to determine whether the experimental values of *LpRT* and Δc are correct.

## Appendix C Implications/interpretation of observed distribution of marker concentrations following injection into cisterna magna

When a bolus dose of an MRI-detectable extracellular fluid marker such as gadobutrol is injected into the cisterna magna of a rat or mouse its concentration in the basal cisterns increases rapidly to a peak at first and then decreases, becoming undetectable after a few hours (see e.g. [443–445]. Very little signal from marker is seen in the subarachnoid space overlying the cortex. In humans following injection into the lumbar sac, there is some delay with a peak in concentration in the basal cisterns within a few hours followed by a decrease over a day [57, 69, 446] (see note added in proof). However in the dorsal subarachnoid spaces near the vertex, the concentration reaches its peak only after about 10 h at a time when the concentration in the basal cisterns is decreasing. Indeed, the marker is still present near the vertex days later when it has become undetectable in the basal cisterns.^51^ These data can be interpreted in terms of a range of kinetic models as illustrated schematically in Fig. 14.

The flow model in column A has been used until recently to describe the human situation. In this model, the marker is presumed to be carried by net flow of CSF from cisterna magna to other basal cisterns and thence via the pial perivascular spaces (see Fig. 3) to the dorsal subarachnoid spaces. It finally leaves the brain via the arachnoid villi to venous sinus blood (or in simple extensions of this model to meningeal lymphatics). In all sub-human species in which alternative routes of elimination have been tested, a large proportion of the bolus dose never reaches the dorsal subarachnoid spaces (see Sect. 3.3) which is inconsistent with this explanation.

An alternative two-compartment pharmacokinetic model that can be used to explain some features of the data for rodents is shown in column B. In this model, the basal cisterns including the cisterna magna and upper parts of the spinal subarachnoid space are regarded as the central compartment and the dorsal cranial subarachnoid spaces as the peripheral compartment. The marker is presumed to be eliminated primarily via the extracellular spaces of cranial nerves leading out of the brain from the basal cisterns and at spinal nerve roots. It only slowly enters and leaves the dorsal subarachnoid spaces. Elimination via the arachnoid villi is presumed to be negligible. So long as the concentration in the basal cisterns exceeds that in the dorsal subarachnoid spaces the concentration in the latter increases. At later times (in pharmacokinetics called the terminal phase of elimination) the concentration in the central compartment decreases so that it becomes less than that in the peripheral compartment.

A third model that allows elimination from both the basal cisterns and the dorsal subarachnoid spaces is indicated in column C. Persistent concentration of marker in the dorsal subarachnoid spaces will be seen whenever the sum of the rate constants for removal from these spaces is substantially less than the sum of the rate constants for removal from the basal cisterns.

Because these different kinetic schemes can all explain longer persistence of marker concentration in the dorsal subarachnoid spaces than in the ventral spaces, that observation of persistence alone cannot argue for the primacy of one outflow route over another. In humans, the proportion of outflow that occurs via arachnoid villi remains unclear.

## Abbreviations

ACSV: Aqueductal CSF stroke volume
ADC: Apparent diffusion constant (in MRI measurements)
AQP4: Aquaporin 4
NVH: Normal volume hydrocephalus
BIH: Benign intracranial hypertension
*c*_*i*_: Concentration of i^th^ solute
cLN: Cervical lymph node
*C*_physio_: Physiological compliance
CM: Cisterna magna
CNS: Central nervous system
CSF: Cerebrospinal fluid
DESH: Disproportionately enlarged subarachnoid-space hydrocephalus
DTI-ALPS: Diffusion-tensor analysis along perivascular spaces
ETV: Endoscopic third ventriculostomy
*ICP*: Intracranial pressure
iIH: Idiopathic intracranial hypertension
iNPH: Idiopathic normal pressure hydrocephalus
iPAD: Intramural periarterial drainage
ISF: Interstitial fluid
*ISFP*: Interstitial fluid pressure
*J*_*v*_: Volume flux [units vol/(area × time) = m^3^/(m^2^ s))
*L*_*p*_: Filtration coefficient
MRI: Magnetic resonance imaging
NPH: Normal pressure hydrocephalus
OB: Olfactory bulb
ONB: Olfactory nerve bundle
OSN: Olfactory sensory neuron
*P*: Pressure
*R*: Universal gas constant
*S*: Stress
*STS*: Solid tissue stress
*T*: Absolute temperature
tMIP: Time maximum intensity projection
tPA: Tissue plasminogen activator
*∆P*_*m*_: (Total) pressure difference
*∆π*: Osmotic pressure difference
*∆V*_*m*_: Cell membrane potential
ϕ: Fraction of a porous solid occupied by the pores (or interstitial spaces)
σ_i_: Reflection coefficient for the i^th^ solute
ν_i_: Number of particles added to solution by i^th^ solute

## Footnotes

1 The volume of blood within the boundaries of the parenchyma has been reported to be about 4 mL (100 g)^-1^ [447–450] which corresponds to a volume of approximately 0.04 x 1160 mL = 46 mL. The volume of blood in the venous sinuses, which are within the skull but outside the boundaries of the parenchyma, is ~ 12.5 mL (a plateau value on a plot of volume versus age for children [451]) for a total blood volume within the skull of ~ 58 mL or ~ 4 % of the total volume. Direct measures of the total volume using labels for blood components at known concentrations are consistent with this percentage [448, 452–454].

2 The volume of CSF within the cranium is not easy to determine. Indeed until the advent of MRI methods there was apparently only one serious attempt. In 1915 Weston [455] drained CSF from recently dead cadavers, employing suitable manipulations of the body to encourage flow. His value for the total volume, 140 mL, has been quoted and requoted frequently, sometimes with incorrect citation (see e.g. [7]) but more frequently with none. Given Weston's methodology, his value is almost certain to be an underestimate. The global mean was 140 mL, but many values were above 180 mL and some even above 300 mL. It should be noted that these values will not include the volume of CSF taken up into perivascular spaces and parenchyma shortly after death (see [456] and Sect. 3.1.5). More recent estimates of the intracranial and vertebral CSF volumes have been determined using MRI to measure the spaces filled by CSF [457]. There is a fundamental difficulty in that some of the dimensions of these spaces are smaller than the resolution, typically ~ 0.5 mm, of the images. Several early measurements are excluded from this list for that reason (e.g. [457]). For the relatively simple geometry of the spine, three prominent values for CSF volume are 74 mL [458], 97 mL [459] and 84 mL ([460]. For the intracranial CSF volume, the range of estimates is wider: 164 mL (supratentorial) [461]; 107 mL (age ~31) and 179 mL (age ~70) [462]; 253 mL [463]; 160 at age 20 increasing to 300 by age 55 [464]; 238 mL [458]; 238±25 mL [243]; 180 mL [465]; 332±112 mL [330, 466]; 255 mL [6]; 304 mL [467];179.1 mL (range 77.2–287.4 mL) ([460] using methods of [468]); 316±11 mL ([469] using CAT toolbox [470]); 161 mL (supratentorial) [471]; and 265 mL age 20-30 yr and 488 mL age >80 yr [472]. De Vis et al [6] and Chazen et al [460] describe some of the difficulties. Estimates for the volume of CSF in the ventricles are: 20.6 mL (lateral ventricles only) [473], 17 mL (age ~31) and 27mL (age ~70) [462], 31 mL (lateral and IIIrd ventricles) [463], 33 mL [474]; 26 mL [458]; 19±7 [243]; 16 mL [465]; 62 mL [466]; 36 mL [467]; 20 mL age < 60 yr [472]. It would, of course, add to confidence if an independent method could be found to confirm the volumes determined using MRI. The round value for the cranial CSF volume used in Fig. 2, 250 mL, is near the middle of the MRI estimates. With 250 mL in the cranium and 85 mL in the vertebral column the estimate for the total volume of CSF is 335 mL which is substantially larger than stated in textbooks.

3 There has been a report that iron oxide nanoparticles leave the brain across the cribriform plate to the nasal mucosa, then reach the pharynx and thereby the gut [118]. If this is confirmed and the route is used by other substances, it might explain the failure in previous studies to account for the entire amount of injected marker substances by the amounts that reach blood and lymph.

4 Claims to the contrary persist in the literature (see e.g. [475–477]) but do not stand close scrutiny as discussed at length elsewhere (see section 2.6 in [1] and [478]). The principal errors that have led some to this position are the failure to distinguish between tracer fluxes of water and net flow of a fluid and attempts to use labelled water (e.g. tritiated water) to trace flow in tissues or small blood vessels (see footnote a in [110]).

5 Because most techniques are invasive, there have been relatively few measurements of CSF production rate in humans and, until recently, these have been only when the invasiveness could be justified for therapeutic reasons. There have been two estimates of total CSF production rate within the ventricles by the perfusion method first introduced by Heisey et al [191], 0.37 mL / min^-1^ [479] and 0.35 mL min^-1^ [192]. Total production has also been estimated by measuring the rate at which CSF could be continually collected from a lumbar drain at low pressure, 0.4 mL min^-1^ [32]. An estimate of CSF production within the lateral and III^rd^ ventricles, 0.137 mL min^-1^ [26] was obtained by measuring the half-time for washout of metrizamide (also known as Pantopaque) and the volume of the ventricles. On this basis, it was concluded that either secretion by the choroid plexus in the IVth ventricle or extrachoroidal production of CSF exceeds the secretion into the lateral and IIIrd ventricles. Subsequently it has been possible to measure CSF flow rate through the cerebral aqueduct as a function of time using various magnetic resonance imaging protocols. These methods have revealed that there is flow in the direction from IIIrd ventricle to IVth during cardiac systole and a similar flow in the reverse direction during diastole. The net flow from the IIIrd to the IVth ventricle, which should equal the CSF production rate in the lateral and IIIrd ventricles can then be calculated as a suitable time averaged value; 0.48 mL min^-1^ [72], 0.4 mL min^-1^ [73], 0.34 mL min^-1^ in daytime and 0.61 mL min^-1^ at night [480] and 0.6 mL min^-1^ [481]. It is difficult to obtain accurate values for this net flow because, in effect, it is the small difference between larger measured quantities, however, the measurements all suggest that the CSF production rate in the lateral and III^rd^ ventricles is similar to or may even exceed the older estimates for the total rate in all of the ventricles. The balance of current evidence thus favours a relatively minor role for the choroid plexus in the IVth ventricle in CSF secretion in man, perhaps consistent with its relative weight.

6 Tight junctions form "tight" links between the cells in epithelial and some endothelial layers. They are normally found in bands around an entire circumference of each cell such that small solutes and water that cross the layer without entering the cells, i.e. via the paracellular route, must pass through the bands. Tight junctions are usually described as either "leaky" or "tight" if they provide low or high resistance respectively to such paracellular movements. The leakiness of the tight junctions in any particular cell layer is determined primarily by which specific claudins are present.

7 In the absence of imposed osmotic gradients active choroid plexus secretion accounts for a large proportion, probably most, of the *net* flux of water into the brain from blood. However, this net flux is dwarfed by the very much larger *unidirectional* fluxes of radiolabelled water that have been measured crossing the blood-brain barrier. The magnitudes of the influx and efflux across this barrier greatly exceed the difference between them, i.e. the net flux. This is as expected because at least semi-quantitatively passive unidirectional fluxes are proportional to the total concentration of water on each side of the barrier, ~55M, while the passive net flux is proportional to the difference between the concentrations on the two sides, ≲ 1mM, which is 55,000 times smaller. There is thus plenty of scope for the active secretion by the choroid plexuses to be larger than the net flux across the blood-brain barrier (whether passive or active) while at the same time being very much smaller than the passive unidirectional fluxes across that barrier measured using for instance tritiated water. For further discussion and references see section 2 in [1] and Footnote a in [110].

8 A detailed consideration of the relative permeabilities of the blood-brain barrier, the endfoot layer, and a slab of parenchymal tissue of the same thickness as the endfoot layer has been presented by Koch et al [482]. It should be kept in mind when considering these comparisons that the diffusion distances in the interstitial spaces are normally considerably greater than the thickness of the endfoot layer.

9 It is almost inevitable that there will be some movement of water accompanying the various forms of transport for ions and other solutes across both the choroid plexuses and the blood-brain barrier (see [25, 31, 483, 484] but see also [485] and for discussion [2, 21]). However, there is no reason to expect that the amount of water so transported will be that required for osmotic balance. At present such transport does not appear to be sufficient to disturb the balance of osmolalities across the blood-brain barrier (see Appendix B and Footnote 17 in [2]) even though the endothelial cells of the barrier do not appear to express aquaporins. However, as the water permeability required is small, it is very likely that no special proteins are needed [2]. By contrast, aquaporins or some other means for increasing membrane water permeability are required in other specialized tissues for instance: a) in renal cortical collecting tubules where there is a need for large, regulated net water fluxes; b) in renal collecting ducts which need only modest fluxes but with small osmotic gradients; or c) possibly [486] in astrocyte endfeet where only small amounts of water need be transported but in a very short time). The renal inner medullary collecting duct provides a striking example of the consequences of the water permeability of the rest of a membrane holding aquaporins. The volume flux of water across the duct wall may even be greater in diuresis when the aquaporin route is shut but the osmotic gradients are large than in antidiuresis when the aquaporin route is open but the osmotic gradients small (see [487], Figs 8.1 and 8.2 in [488] and the section “The MCD produces a concentrated urine by osmosis, driven by the osmotic gradient between the medullary interstitium and the lumen” in [489]).

10 The magnitude of the net flow of CSF through any constriction in the flow pathways is the difference in size between the much larger forward and backward movements which constitute the pulsatile flow. However, if the net flow is to be calculated, the sizes of the forward and backward movements must be known to high percentage accuracy. Largely for this reason, MRI-PC based calculation of net flow has been successful only for the flows through the cerebral aqueduct [82] For further discussion see [110].

11 Solute transport in the interstitial spaces of grey matter is primarily by diffusion (for references to the extensive literature and discussion see [4, 10, 490, 491]. Any conceivable level of flow through the interstitium (i.e. that which could be supplied by flow in the perivascular spaces and/or secretion across the blood-brain barrier) will produce flow velocities that would not detectably bias the diffusion of solutes towards or away from either arterioles or venules [492] as was required in the original glymphatic hypothesis [4, 94]. While this view is not universally accepted (see [92, 493–495]), it is not contradicted by any experimental data, in particular not by the published MRI studies (see e.g. [443]). The studies indicating that both diffusion and convection are needed to explain solute movements within the parenchyma define parenchyma to include perivascular spaces, basement membranes and walls of the arterioles, venules and microvessels. In these, flow is, indeed, likely to be important.

12 The reasons why the blood-brain barrier of parenchymal microvessels is not in this list have been discussed in Sects. 3.2 to 3.2.1.

13 Solutes that reach the nasal mucosa can leave it via lymph [111, 136, 496], via blood-flow [141], and according to one recent report via mucous flow to the back of the throat where they can be part of the fluid swallowed and delivered to the gut [118].

14 Carare, Weller and colleagues have promoted the iPAD hypothesis, that the extracellular spaces of the smooth muscle layers of arterial walls provide an efflux pathway for solutes from the parenchyma [95, 103, 104, 380, 497–500] (see sections 5.2.2 and 5.2.3 in [4] for further discussion). An attractive feature of this pathway is that it would provide a route for outflow of fluid (albeit of unknown resistance) from the parenchyma to the basal cisterns and/or lymphatics at the base of the skull.

15 As suggested by one of the referees for this review, a simple scaling argument suggests that humans will need to have additional routes for CSF outflow compared to rats. The ratio of the volume (or weight) of the human brain to that of a rat is approximately 1400 g / 2 g = 700. If CSF production subserves the functions of the brain, this production will scale in proportion to brain weight and this is (approximately) observed, see e.g. Table 6.2 in [7]. By contrast it is not unreasonable to expect outflow via a route such as the extracellular space of a nerve to scale in proportion to the cross-sectional area of the nerve which in turn will scale proportionally to a rough approximation to the surface area of the brain excluding all the infoldings. This area varies as the 2/3rds power of the volume and thus as the 2/3rds power of the weight. Hence comparing humans and rats, the ratio would be (700)^2/3^ ~ 80. If indeed the conductance (or permeability) of the outflow routes does scale with this area, this means that either the pressures driving outflow would need to be 700/ 80 ~ 9 fold greater in the human or that there are additional routes for outflow. The pressures are similar. Thus, even though this calculation is based on many questionable assumptions, it does suggest that outflow routes additional to those important in rodents are likely to be needed in species with big brains. To put it another way, there is a prima facie case that the cribriform plate will not suffice as a a dominant route for outflow in humans. There is also indirect evidence discussed in Sect. 3.4.3 that there are pathways for elimination sensitive to sagittal venous pressure. Extrapolation across species must be done with care. Direct evidence is needed.

16 See footnote 25 in Sect. 5.4 for a caveat.

17 Note that the changes in horizontal dimensions occur without any change in the horizontal stress. Not only can the stress be different in differing directions, but the stress can also produce changes in dimensions in more than one direction. Furthermore stress, unlike pressure, can also act parallel to a surface as well as perpendicular to it. The parallel component is called a sheer stress. Sheer stress is critical in brain trauma as it is sheer rather than compression which tears the brain and vasculature.

18 Terzaghi's formulation has become standard in engineering applications because to an excellent approximation the volume, mechanical properties and water content of the porous solid are not affected by hydrostatic pressure [501]. Skempton [170] discusses Terzaghi's concept of effective stress and gives some specific examples, e.g. that sediments on the sea floor have the same structure and the same water content regardless of depth even though the hydrostatic pressures applied by the sea water can be very different. In Terzaghi's formulation the hydrostatic pressure acts on both the fluid and the framework, but the effective stress acts only on framework [501]. Thus the only stress applied to the fluid in the pores is the interstitial fluid pressure, *ISFP*. This pressure has had various names including: i) pore water or pore fluid pressure (Terzaghi (1923) as translated in [171])[338, 363], ii) neutral pressure [501] and iii) interstitial fluid pressure [173]. The last has the advantage of referring to the fluid present in biological tissues, ISF, and so is used in this review. The remaining part of the total stress in Terzaghi's formulation is Terzaghi's effective stress, *ES*, defined by *ES* = *S* - *ISFP*. Thus somewhat confusingly even though the effective stress is taken to act only on the framework it is calculated as if, like *S* and *ISFP*, it acts on the entire area of the porous solid.

19 Consider a small cubic region of the porous solid with the area of each face equal to *A*. As Biot [172] has shown, ϕ is also the fraction of *A* that is intersected by the pores. The force exerted at this face by the fluid within the pores (and thus the force exerted on the fluid within the pores) is then *F*_pore_ = *ISFP* x ϕ *A*. Similarly, the force exerted at this face by the framework is *F*_frame_ = *STS* x (1- ϕ)* A*. But the sum of these forces is the total force exerted on the face which is just the stress times the area. Thus

$$S{\text{x}}A \, = \, ISFP{\text{x}}\phi A + STS{\text{x }}({1} - \phi )A$$

and

$$S = \, ISFP{\text{x}}\phi + STS{\text{x }}({1} - \phi ).$$

20 Because *S* and *ISFP* have the same meanings in both Terzaghi's and Biot's formulations, the relation between *ES* and *STS* is simply derived. Thus because

$$S = ISFP + ES \left( {{\text{Terzaghi}}} \right)$$

and

$$S \, = \, ISFP{\text{x}}\phi + STS{\text{x }}({1} - \phi )\left( {{\text{Biot}}/{\text{Guyton}}} \right) \, ,$$

the right-hand sides of these expressions can be set equal to each other and solved for the solid tissue stress,

$$STS = ISFP + ES \, /({1} - \phi ).$$

When there is no component of imposed stress that acts solely on the framework, *ES* = 0 and *S* = *ISFP* = *STS,* which, in words, says that under these conditions the stress is a pressure. This relation also makes clear that in Biot's as well as Terzaghi's formulation the hydrostatic pressure equal to *ISFP* acts on both the liquid and framework components of the porous solid. Indeed this and the incompressibility of the liquid and solid components of the porous solid are the primary reasons why the hydrostatic pressure does not produce changes in the structure, dimensions and fluid content of the porous solid. Regardless of whether stresses are calculated using Biot's or Terzaghi's formulation, it is necessary to know the fraction of the porous solid that is fluid in order to interpret measurements of the resistance to fluid flow. Thus the fact that Biot's formulation involves an additional parameter, ϕ, is not a serious drawback in practice. It should be noted that neither formulation considers any hindrance to fluid movement at the surfaces of the porous solid (e.g. by a membrane) or fixed charges that are part of the framework. Consideration of either of these may be possible by introducing local osmotic pressure. This is not required for the discussions of ventriculomegaly and hydrocephalus. Fixed charges are considered further in the discussion of the Donnan effect in [34].

21 *ISFP* cannot be measured by simply inserting a fluid filled needle into a tissue and connecting it to a pressure transducer [173] but it can be measured using several more elaborate techniques. In the gold-standard method for measuring constant *ISFP*, sufficiently large hollow porous capsules are implanted and the pressure on the free fluid that accumulates inside measured using a transducer [502]. More conveniently, with less damage to the tissue, and with better time and spatial resolution, *ISFP* can be measured as the pressure on free fluid in the barrel of a pipette with the tip plugged with a wick (often one or more cotton fibers) [503] [504]. Even better time and spatial resolution and even less damage to the tissue can be achieved with a micropipette with a very small tip by using a servo-null method to measure the pressure on the fluid within the barrel (see e.g. [505]).

22 There are qualifications and exceptions to the statement that there can be no gradients of total pressure. Firstly the discussion in the main text ignores gravity and centrifugal force. For instance when a person is standing the pressure in the lumbar sac will be greater than that in the lateral ventricles by an amount, ρ*gh*, where ρ is the density of CSF, *g* is the acceleration resulting from gravity, and *h* is the vertical distance between the ventricles and the lumbar sac (see e.g. [7, 506]). This difference in pressure is just that which balances gravity so that there is little or no net force acting to move the fluid upwards or downwards. How ventricular pressure varies with posture is somewhat complex, see e.g. [185–187], but the difference between the lumbar and ventricular pressures is well described by ρ*gh*. Secondly, whereas normally the total stress is the same throughout the brain and spinal cord (except for the effect of gravity), there are pathological conditions in which CSF flow is obstructed *and* the parenchyma is constrained from moving thus allowing development of total stress gradients. One important example occurs when the connection between the CSF containing spaces in the cranium and the vertebral subarachnoid spaces is blocked by protrusion of the hindbrain and/or cerebellum into the foramen magnum (Chiari malformation). This block can allow a substantial difference in total pressure between the cranium and the vertebral subarachnoid spaces. The effects of oedema on total pressure and herniation of parts of the brain through the tentorium or foramen magnum are considered in [34]. It is likely that the major constraints imposed on the shape and position of the outer boundary of the parenchyma occur by its contacts with the meninges and thereby the skull. However, it should be noted that the parenchyma itself can support small pressure differences across its structure. Levine [385] and others have considered the constraint that would occur if the parenchyma were a spherical shell completely enclosing a spherical ventricle (except for a hole representing the aqueduct). Enlarging the ventricle will deform the shell forcing it to have larger internal radius and larger ventricular surface area. Thus it becomes stretched circumferentially and compressed radially. This deformation can in principle support a pressure difference between the inside and outside surfaces of the shell somewhat analagous to the pressure difference across the wall of a balloon. The calculations are complex even for models with grossly oversimplified geometry and furthermore those that have been done do not allow for steady-state flux of ISF through the parenchyma.

23 The outflow resistance can be determined directly from its definition if CSF can move freely about the brain and measurements can be made of both *ICP* and the outlet pressure. If the outlet pressure is constant, i.e. it doesn't vary with the pressures and volumes within the brain [194], the resistance can be calculated without knowing the outlet pressure by infusing fluid at a constant rate into some part of the CSF containing spaces (see e.g. [193]), waiting until the pressure reaches a plateau, measuring the increase in *ICP* and calculating

$$\left( {\text{outflow resistance}} \right) \, = \, {{\left( {{\text{increase in}}ICP} \right)} / {\left( {\text{infusion rate}} \right)}}.$$

However, it should be noted that increases in *ICP* may compress structures in the dura including the venous sinuses and probably meningeal lymphatics which may, contrary to Davson's assumption, increase the outlet pressures for at least some of the routes of outflow [15, 507]. There is also another caveat. The resistance determined by infusion is that which applies to routes from the sites of infusion to the destinations of the routes of outflow for the fluid infused. That will not necessarily be the same as the resistance between the choroid plexuses in the ventricles and the destinations for that outflow. Obstruction of CSF flow may affect these resistances differently. A blatant example of this effect occurs with aqueductal stenosis if the infusions and pressure measurements are in the lumbar sac. It is not clear whether the dependence of the calculated outflow resistance on the site of the infusion can explain some of the failures of outflow resistance measurement to predict shunt responsiveness [390]. It is also possible to estimate the outflow resistance from measurement of the time course of the pressure change after a bolus injection of fluid. Because the change in *ICP* is then short-lived this procedure may partially avoid the complications of variations in outlet pressure with *ICP*. However, set against this advantage, interpretation of the time course of pressure after a bolus injection involves moderately complex calculations using a model for how the pressure varies with the volume of CSF present (see [195]).

24 Throughout this review, the circulation of CSF (net flow) and its outflow from the brain are treated as passive processes, driven by pressure gradients. This is not entirely correct. For instance, *net* movements of CSF through the ventricles are affected to some extent by the beating of cilia on cells of the ependymal lining [508, 509]. The effects of cilia are largest close to the lining ependymal layer where they are located relative to those throughout the volume of the CSF containing spaces [510]. The effects of cilia are thus expected to be more marked in small animals where most of the volume is close to the surfaces. At present there is little evidence that disorders of cilia account for more than a few, rare instances of hydrocephalus in humans [511]. One of the sources of propulsion of CSF along perivascular spaces is thought to be variations in size of the blood vessels [17, 512, 513]. These arise from changes in blood volume within the vessels during the cardiac and respiratory cycles, vasomotion and neurovascular coupling [66, 101–108, 512, 514] (for discussion and further references see section 5.2.4 in [4] and sections 9.3.2, 9.4.2 and 9.5.2 in [92]). A complete description of CSF movements should include all factors propelling such movements.

25 The relation between a volume of CSF added and the change in *ICP* it produces is described by the compliance of the cerebral contents, defined as the volume added divided by the increase in pressure. The compliance depends on how much time intervenes between the volume addition and the pressure measurement because volume changes in the parenchyma corresponding to movements of ISF take time to occur (see e.g. [515]). The compliance is also a highly non-linear function of the volume of CSF present. As Marmarou et al [147] noted, when fluid is injected into the subarachnoid spaces, the compliance is determined in part by the pressures needed to reduce the volume of blood (or as he stated, the volume of the vascular elements) within the cranium. Similarly, the pressure increases required to insert more blood into the brain during systole are to a large extent just those required to expel CSF from the cranium into the vertebral subarachnoid spaces. In either case small additions can be accommodated easily by these shifts so the increase in *ICP* after compensation occurs is small and the compliance is large. However, blood volume is only about 4% of the total, and only so much fluid can be shifted from the cranium into the spine and thus large increases in CSF volume cannot be easily compensated, the pressure increase is then large and the compliance becomes small. This is sometimes described by saying that the compensated *ICP* increases exponentially with CSF volume or equivalently that the logarithm of *ICP* increases linearly with CSF volume [516]. Treating the components of the parenchyma as if they are incompressible is a good first approximation, but it may be possible to measure deviations from incompressibility. A "physiological compliance" [517] can be calculated from the amplitude of the variation in *ICP* during the cardiac cycle if it is possible to measure sufficiently accurately the inflows and outflows of both blood and CSF from the head,

$$C_{{\text{physio}}} = \, \left( {\text{total change in fluid volume within the head}} \right) \, / \, \left( {{\text{change in}}ICP} \right).$$

Balédent et al [518] have reported in an abstract that this compliance can be determined and is 6-7 fold smaller than the compensated compliance determined by slow infusion of CSF. Some authors refer to the response of the brain at constant composition as the

$${\text{elasticity }} = { 1}/ \, \left( {\text{instantaneous compliance}} \right)$$

and the response when the amount of blood or CSF is allowed to change as the

$${\text{elastance }} = { 1 }/ \, \left( {{\text{compliance}}} \right)$$

where the pressure change is measured after the compensating fluid movements have occurred. The elasticity is expected to be substantially larger than the elastance (see e.g. [519]). The physiological compliance should be intermediate between the two. It should be noted that both elasticity and compliance are measured on time scales that are assumed to be long compared to the time for CSF shifts between the head and vertebral subarachnoid spaces but short compared to the time taken for the quantity of *ISF* in the parenchyma to change. Those slower changes, which may be important in parenchymal shrinkage in hydrocephalus, are considered in Sects. 9.2.1.2 and 9.2.2.3.1.

26 See [173]for discussion of why it is not sufficient to insert fluid filled tubing into a tissue and measure the pressure at the far end. See also Footnote 21.

27 Intracranial hypertension without obvious cause has been given many names including: "increased intracranial pressure without brain tumor, pseudotumor cerebri, pseudotumor cerebri syndrome" , "normal volume hydrocephalus" (NVH), "idiopathic intracranial hypertension" (IIH) and "benign intracranial hypertension" (BIH) [158, 206, 209, 222, 520–522]). Calling it BIH is objectionable to some because its consequences, even when these are just chronic headaches, are hardly benign. NVH is an unfortunate term because hydrocephalus is by definition not normal volume. Advocates of "pseudotumor cerebri syndrome [158] point out that it "makes no assumptions about etiology or mechanism". However, iIH has an advantage in that it refers directly to "intracranial hypertension". Despite "idiopathic" ceasing to be appropriate as causes are elucidated [206], iIH is the name in common use.

28 Weight-reduction is the first and sometimes successful measure employed to reduce *ICP* and the headache, which was often the presenting symptom [523]. Bariatric surgery is also employed [524, 525]. Neurosurgical intervention is required when vision is imminently imperilled [526].

29 There have been suggestions that the route of outflow of fluid from the brain that is defective in iIH is outflow from the parenchyma via extravascular preferred routes for flow of ISF (including glymphatics), leading to arachnoid villi that in turn lead to either meningeal lymphatics or venous sinuses [160, 527, 528] However, reducing fluid removal from the parenchyma (which presupposes that such removal occurs normally) rather than from the subarachnoid spaces would be expected to lead to parenchymal swelling which is not an essential feature of iIH. Recently Schartz et al [529] found using DTI-ALPS (diffusion tensor image analysis along the perivascular space) that flow along glymphatics is depressed in iIH. It should be noted, however, that use of DTI-ALPS to assess glymphatic function is at present controversial [530, 531].

30 The prediction that there will not be any volume change depends critically on there being no direct route by which *ISF* can leave the parenchyma to a destination which has a fluid pressure less than *ICP* (see Sects. "9.1.3 and 9.2.2.3.1). If there is such a route from the parenchyma, increase in the outlet pressure of a route leading from the subarachnoid spaces would be likely to lead to increased volume of the CSF containing spaces (as observed by Alperin et al [243]) but reduced parenchymal volume.

31 In addition to the potentially large effect considered in the previous footnote, there may be small, probably imperceptible, changes in the volumes of the ventricles, parenchyma and subarachnoid spaces reflecting both the small but finite compressibility of the liquid and solid components of the cerebral contents, small changes in the size of the skull [249] and the change in the volume of blood within the confines of the parenchyma and subarachnoid spaces (see footnote 25). Blood volume could increase or decrease depending on arterial blood pressure and vascular tone as well as *ICP* [532].

32 For a different perspective on the importance of Dandy and Blackfan's results see [246]. For discussion of some of Di Rocco's results see Sect. 9.2.3.

33 "Pediatric" is usually taken to include children up to the age of puberty. The important distinction here is the fusion of the brain sutures and closure of the fontanelles which happens at about 18 months. In this review pediatric hydrocephalus refers to hydrocephalus in fetuses and infants.

34 Ventriculomegaly in foetuses can be seen in routine ultrasound scans, which aids its early diagnosis. By contrast increased volume of the subarachnoid spaces would be more diffuse and thus difficult to detect.

35 See Sects. "5.5 and 9.2.1 for discussion of the difference between total and fluid pressures in shrunken or collapsed subarachnoid spaces. The subarachnoid space total pressure can be equal to ICP while the fluid pressure is less because contact between the parenchyma and the meninges increases the solid tissue stress.

36 In cats with a closed skull, kaolin produced a hydrocephalus with enlarged but stable ventricles. The route for outflow from the ventricles that balanced CSF production and allowed stable ventricular volume was apparently from the IVth ventricle via the central canal of the spinal cord [331, 533, 534].

37 An impressive video of the view from the endoscopy tool showing a repeated ETV following formation of a new obstruction to CSF flow is available as supplementary material to [308].

38 In rats with kaolin-induced [53, 329] or N-butyl cyanoacrylate-induced [328] hydrocephalus, the normal route of CSF exit is blocked but ^14^C-sucrose and ferritin, which presumably need an aqueous flow pathway to escape, can still leave the ventricles [53, 328, 329]. In contrast in rhesus monkeys with non-communicating hydrocephalus induced by inflating a balloon in the IVth ventricle, phenolsulphophthalein was retained in the ventricles for more than 5 h suggesting that there was no new pathway or escape of hydrophilic solute. In a single experiment [330], iohexol injected into a lateral ventricle of a hydrocephalic patient was seen to reach the ambient cistern after only a short delay arguing that the apparent openings observed in the MRI were patent.

39 Evidence for this has been obtained: by observing tissues fixed at various stages of the development of hydrocephalus [253, 256, 261, 535], by measuring the difference in wet and dry masses of the tissue and calculating water content [333, 350, 351], by reduced density in CT scans [280], and by calculation of relative amounts of water from MRI images [356, 536]. (For further discussion and references see [250],^339^] and for aspects relevant to the CNS [37, 340, 341] with further discussion in [4, 92]). Secondly something must keep the interstitial fluid pressure (see Sect. 5.2.2).

40 It is very tempting to suggest (see section 5.6 in [3]) that the distance from the periventricular surface to the perivascular spaces determines the depth of periventricular oedema.

41 There is also good evidence that large solutes, e.g. radioiodinated serum albumin, injected into the parenchyma are cleared from it to CSF and/or lymph [97, 496, 537].

42 At least in principle the reduction in volume of cortical subarachnoid spaces could occur with block of normal access routes of CSF to these spaces from the ventricles and basal cisterns, block of the usual routes of outflow from the spaces and outflow of CSF via the parenchyma. There appears to be no evidence for or against the occurrence of this scenario.

43 Deo-Narine et al [538] injected a silastic mixture into the prepontine cisterns using a catheter introduced via the cisterna magna. With their technique in which silastic spread as far as the vertebral subarachnoid spaces, the blockage prevented passage of a marker, Gd-DTPA, from the ventricles to the vertebral subarachnoid spaces. They described the resulting hydrocephalus as non-communicating. The reason why Deo-Narine et al and James et al could produce different patterns of block, in each case reproducibly, is likely to be the difference in the methods used for the silicone injection (compare [146] with [538]. James et al were at pains to avoid non-communicating block. Deo-Narine et al used the reduction in outflow from the ventricles that they produced to allow study of marker penetration into the parenchyma from the ventricles and thus will have had little incentive to reproduce the earlier results of James et al.

44 The idea is that with less compliance, when blood is pushed into the arteries in the subarachnoid spaces the opposing pressure restricting enlargement of the arteries is greater. Thus: the arteries expand less; less CSF is expelled from the cranial subarachnoid spaces into the vertebral subarachnoid spaces; more of the blood pressure change is transmitted to the blood vessels in the parenchyma; the pressure changes in the parenchyma and ventricles are larger; and more CSF is expelled from the ventricles into the veretebral subarachnoid spaces. All of these effects are observed experimentally, but they do not establish that the change in compliance is the cause of the hydrocephalus.

45 Support for pulsatility playing an important role in the development of ventriculomegaly was also provided by studies of Di Rocco et al [539–541] (see [542] for a pedagogic description) who inserted small balloons into the lateral ventricles and then periodically inflated and deflated them. However, it is now known that the pulsatility arises not from changes in the choroid plexuses, which the balloon-induced changes were designed to mimic, but rather from changes in all the microvasculature of the parenchyma, see Sect. 3.3.1). Furthermore, the balloons almost certainly were in direct contact with the walls of the ventricles and thus effects other than simple pressure changes may have occurred.

46 The dementia in iNPH ha been said to be of the "subcortical type resembling that seen in frontal disorders: forgetfulness, inertia, inattention, decreased speed of complex information processing, and impaired ability to manipulate acquired knowledge" [240]. Vanneste was citing [543] where it was asserted that hydrocephalic dementia could be distinguished from Alzheimer's dementia by "psychiatric and psychometric evaluation". However, present diagnostic criteria do not appear to rely on such testing. Given the relatively poor success of using any other criteria, should such testing be reconsidered, perhaps as a supplement?.

47 In cases of doubt it is recommended to do a lumbar tap test in which CSF is withdrawn to see if there is any improvement in the symptoms [544, 545]. An alternative is the extended lumbar drainage test in which a larger amount of CSF is withdrawn but over a period of 3 days [546]. The rationale for either test is that if as observed, at least sometimes, shunting can produce rapid improvements in symptoms by reducing CSF volume, then the much less invasive tests that also reduce CSF volume should also produce improvement. Presumably the reasons the extended lumbar drainage test has not been more widely adopted are marginal extra benefit, the increased risk entailed with multiple lumbar punctures and, not least, cost. If phase contrast MRI is available to allow measurement of the aqueductal stroke volume (see Sect. 3.3.1), a good shunt response is predicted if aqueductal stroke volume is at least 42 µL [547], but see also [548]. As a further alternative if ICP monitoring is possible, increased pulse pressure can be used as a criterion instead of increased aqueductal stroke volume [91, 549]. As discussed in Sect. 9.2.3, increased pulse pressure and aqueductal stroke volume are expected whenever cranial subarachnoid space volumes are reduced or flow through them is blocked.

## Note added in proof

Vinje et al. [446] report that “that no more than 33% of gadobutrol injected intrathecally in humans is located within the intracranial compartment at any given point in time” while 25% “of the injected tracers is found within the brain between 6 and 24 h.” They “suggest that a relatively large portion of CSF may be drained from regions outside the cranium”, i.e. that there is substantial absorption from the vertebral subarachnoid space. van Osch et al. [553] in a comprehensive review of kinetic studies with gadobutrol discuss many of the complications involved in analyzing observations to yield parameters like the rates of penetration into and efflux from the cortex.

Eide and Ringstad [554] show that large arteries running in the subarachnoid space surrounding the cortex are covered by a sheath through which gadobutrol penetrates slowly compared to the anterograde progression along the artery. Thus following intrathecal injection of gadobutrol the NMR-visible front of gadobutrol arrives as a torus surrounding the artery with gadobutrol-free surrounding subarachnoid space. Within tens of minutes the gadobutrol spreads into that space. These results are consistent with but, given that entry occurs over hours, do not prove that markers arriving at a region of the cortical surface are directed towards brain entry via the perivascular spaces of the arteries penetrating into the cortex [66, 67, 94].
